# New Pregnane Glycosides from *Gymnema sylvestre*

**DOI:** 10.3390/molecules20023050

**Published:** 2015-02-12

**Authors:** Rui Xu, Yu Yang, Yang Zhang, Fengxia Ren, Jinlong Xu, Nengjiang Yu, Yimin Zhao

**Affiliations:** Institute of Pharmacology and Toxicology, Academy for Military Medical Science, 27 Taiping Road, Haidian District, Beijing 100850, China; E-Mails: xurui2015@163.com (R.X.); tommase2015@163.com (Y.Y.); zyqci2015@163.com (Y.Z.); ren2015victory@163.com (F.R.); xujinlong2015@163.com (J.X.)

**Keywords:** *Gymnema sylvestre*, Asclepiadaceae, pregnane glycosides, α-glucosidase

## Abstract

Four new pregnane glycosides **1**–**4** were isolated from the ethanol extract of the stem of *Gymnema sylvestre* and named gymsylvestrosides A–D. Hydrolysis of compound **1** under the catalysis of *Aspergilus niger* β-glucosidase afforded compound **5** (gymsylvestroside E). Their structures were determined by spectroscopic methods such as HRESIMS, 1D and 2D NMR, as well as HMQC-TOCSY experiment. Compounds **1**–**4** were screened for *Saccharomyces cerevisiae* α-glucosidase inhibitory activity.

## 1. Introduction 

*Gymnema sylvestre* (Retz) Schult is a liana plant of the Asclepiadaceae family that grows in tropical and subtropical regions of the World. In China, it is distributed mainly in the provinces of Guangdong, Guangxi and Yunnan [1] and is traditionally used by local people as an anti-inflammatory and analgesic herbal medicine [2]. It is also a folklore medicine in India used for the treatment of malaria, hyperglycemia, mosquito and snake bites [3]. Pharmacological studies showed that *G. sylvestre* has hypoglycemic [4,5], anti-caries [6,7] and weight reducing [8,9,10] effects. Triterpenoids [11,12,13,14,15,16,17,18], cyclitols [19], flavonoids [20], peptides [21], pectin [22] and alkaloids [23,24] have been isolated from this plant. Some triterpene saponins from *G. sylvestre* were found to be able to attenuate hyperglycemia induced by pituitary growth hormone or adrenocorticotropic hormone [25,26] and to inhibit intestinal glucose absorption in diabetic rats [27,28,29,30].

**Figure 1:**
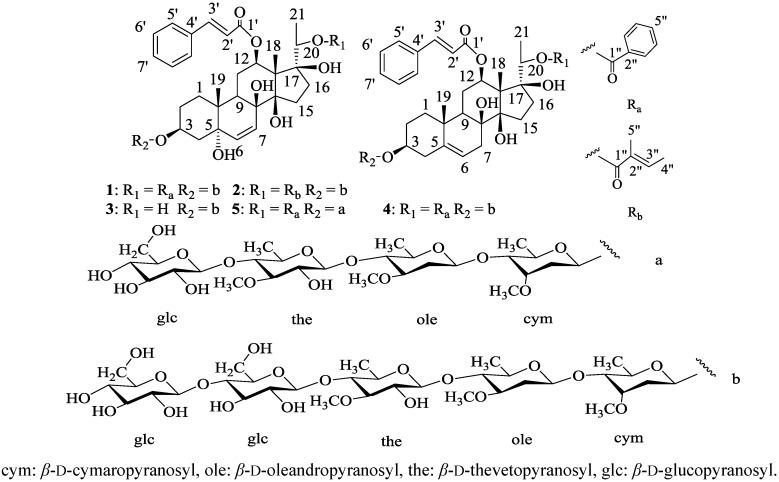
Structures of compounds **1**–**5**.

In the 1990s, Yoshikawa *et al*. reported the isolation of a series of pregnane glycosides from the species *G. alternifolium* [31,32]. In our study on the hypoglycemic constituents of *G. sylvestre*, four new pregnane glycosides **1**–**4** were isolated from the 50% ethanol extract of the dry stem of *G. sylvestre* and named as gymsylvestrosides A–D. The NMR spectra of these compounds showed the presence of a complex sugar moiety comprising deoxysugars and glucose in their structures. Serious overlapping of the NMR signals made it difficult to give a clear assignment of the glycosyl structure. Several enzymes were screened for the ability to hydrolize the glycosyl moiety. The β-glucosidase from *Aspergilus niger* was found to be able to remove a terminal glucose from the sugar moiety of compound **1**. Enzymatic hydrolysis of compound **1** catalyzed by *Aspergilus niger* β-glucosidase afforded compound **5** (gymsylvestroside E), which gave NMR spectra clear enough for the elucidation of the glycosyl structure. The present paper describes the isolation and structural elucidation of gymsylvestrosides A–E (**1**–**5**, Figure 1).

## 2. Results and Discussion

### 2.1. Isolation and Structure Elucidation

The 50% ethanol extract of the dried stems of *G. sylvestre* was partitioned between water and *n*-butanol. The butanol portion was separated successively by column chromatography over D-101 macroporous resin, silica gel, MCI resin and preparative HPLC equipped with an ODS column. Four pregnane glycosides **1**–**4** were thus obtained. Hydrolysis of compound **1** catalyzed by β-glucosidase afforded compound **5**. The molecular mass of **5** was 162 units less than that of **1**, indicating the loss of a hexose group from **1**. Positive results in the Libermann-Buchard and Keller-Kiliani reactions pointed out that compounds **1**–**5** were steroidal saponins and contained α-deoxy sugars.

Compound **1** was isolated as a colorless powder. The positive HRESIMS gave a pseudo-molecular ion peak at *m/z* 1,422.6627 [M+NH_4_]^+^ (calcd. 1,422.6694), corresponding to a molecular formula of C_70_H_100_O_29_. The ^13^C-NMR and DEPT spectra displayed signals of 70 carbons, comprising six methyls, eight methylenes, a methine, three methoxyl groups, two hydroxylmethenes, twenty-six hydroxylmethines (including five anomeric sugar carbons), four oxygenated quaternary carbons and two ester carbonyls.

The carbon signals of two angular methyl groups (δ_C_ 12.4 and 21.5), a methyl group (δ_C_ 15.6) attached to a tertiary carbon (δ_C_ 75.3), four oxygenated quaternary carbons (δ_C_ 74.7, 74.0, 87.7 and 88.2) and a double bond (δ_C_ 136.7 and 127.3) exhibited the features of a polyhydroxypregnane fragment, which was analogous to the pregnane skeleton of the known compound prosapogenin reported in the literature [32,33]. The anomeric carbon signals at δ_C_ 97.7, 101.8, 103.9, 104.5 and 104.9 and the ester carbonyl signals at δ_C_ 165.6 and 166.8 indicated that compound **1** was a pregnane glycoside carrying two acyl groups.

The ^1^H and ^13^C-NMR (Table 1 and Table 2) signals of the pregnane skeleton were assigned by analysis of the ^1^H,^1^H-COSY, TOCSY, HMQC and HMBC spectra. The ^1^H,^1^H-COSY and TOCSY spectra of **1** displayed a spin system from H-1 to H-4, H-9 to H-12, as well as correlations between H-6/H-7, H-15/H-16 and H-20/H-21, which could designated to the four ring skeleton of a pregnane derivative. The HMBC spectrum showed correlations from H-19 to C-1, C-5, C-9 and C-10, H-18 to C-12, C-13, C-14 and C-17, indicating the two angular methyl groups were connected to C-10 and C-13, respectively. The HMBC correlation from H-21 to C-17 and C-20 suggested the attachment of a side chain on C-17. The presence of a double bond between C-6 and C-7 was evident from the HMBC correlations from H-6 (δ_H_ 5.94, d, *J* = 10.4 Hz) to C-10 (δ_C_ 39.6) and C-8 (δ_C_ 74.0) and H-7 (δ_H_ 6.26, d, *J* = 10.4 Hz) to C-9 (δ_C_ 36. 6) and C-5 (δ_C_ 74.7). These data showed a structural feature similar to that of the known compound gymnepregoside E [33]. Further comparison of the NMR data of the two compounds revealed that the NMR signals of the aglycone part of **1** were nearly identical to those of gymnepregoside E. Full assignment of the ^1^H and ^13^C-NMR signals of the pregnane skeleton was realized by analysis of the ^1^H,^1^H-COSY, TOCSY, HMQC and HMBC spectra and comparing with those of gymnepregoside E. The aglycone structure of compound **1** was thus determined as pregn-6-ene-3,5,8,12,14,17,20-heptol.

The relative configuration of the pregnane skeleton was determined by a ROESY experiment carried out in DMSO-*d*_6_. In the ROESY spectrum, the correlated signals between H-1a (δ_H_ 1.59)/H-3 (δ_H_ 3.06) and H-1a/C_5_-OH (δ_H_ 3.59) indicated that the A ring has a chair-like configuration and the substitution on C-3 is β-oriented. The NOE correlation between H-1a/H-9 (δ_H_ 1.85) suggested an A/B trans junction for the A ring. The H-18 signal at δ_H_ 1.48 showed correlations with C_8_-OH (δ_H_ 4.09), C_14_-OH (δ_H_ 5.23) and C_17_-OH (δ_H_ 5.30), indicating that these hydroxyl groups are β-oriented. The configuration of H-12 was confirmed to be α-oriented by the NOE between H-12 (δ_H_ 4.73) and H-9. Judging from the NOEs between H-12/H-20 (δ_H_ 4.61), H-20/H-16a (δ_H_ 1.96) and H-21(δ_H_ 1.21)/H-16b (δ_H_ 1.79), the C-20 was considered to have a *S* configuration.

**Table 1:** ^1^H-NMR (400 MHz) of the aglycones of compounds **1**–**5** (in pyridine*-d*_5_, *J* in Hz).

NO.	δ_H_ (*J* in Hz)					
	1 ^(a)^	1 ^(a),(b)^	2	3	4	5 ^(a)^
**1a**	1.68 (m)	1.59 (m)	1.71 (m)	1.73 (m)	2.44 (m)	1.69 (m)
**1b**	2.18 (m)	1.21 (m)	2.21 (m)	2.22 (m)	2.57 (m)	2.17 (m)
**2a**	2.01 (m)	1.63 (m)	2.01 (m)	2.03 (m)	1.79 (m)	2.02 (m)
**2b**	2.16 (m)	1.26 (m)	2.16 (m)	2.16 (m)	2.08 (m)	2.14 (m)
**3**	4.18 (m)	3.06 (m)	4.18 (m)	4.20 (m)	3.85 (m)	4.20 (m)
**4a**	2.07 (m)	1.79 (m)	2.08 (m)	2.08 (m)	2.41 (m)	2.07 (m)
**4b**	2.25 (m)	1.64 (m)	2.27 (m)	2.27 (m)	2.58 (m)	2.27 (m)
**5-OH**		3.59 (s)				
**6**	5.94 (d, 10.4 Hz)	5.40 (d, 10.4 Hz)	5.93 (d, 10.4 Hz)	5.92 (d, 10.4 Hz)	5.36 (m)	5.95 (d, 10.4 Hz)
**7a**	6.26 (d, 10.4 Hz)	5.63 (d,10.4 Hz)	6.26 (d, 10.4 Hz)	6.26 (d, 10.4 Hz)	2.41 (m)	6.25 (d, 10.4 Hz)
**7b**					2.53 (m)	
**8-OH**		4.09 (s)				
**9**	2.41 (m)	1.85 (m)	2.41 (m)	2.41 (m)	1.78 (m)	2.41 (m)
**11a**	2.20 (m)	1.72 (m)	2.23 (m)	2.24 (m)	2.05 (m)	2.22 (m)
**11b**	2.43 (m)	1.54 (m)	2.49 (m)	2.43 (m)	2.37 (m)	2.45 (m)
**12**	5.38 (m)	4.73 (m)	5.35 (m)	5.37 (m)	5.25 (m)	5.38 (m)
**14-OH**		5.23 (s)				
**15a**	2.00 (m)	1.70 (m)	1.48 (m)	1.48 (m)	1.80 (m)	2.00 (m)
**15b**	2.19 (m)	1.66 (m)	2.17 (m)	2.16 (m)	2.15 (m)	2.21 (m)
**16a**	2.01 (m)	1.96 (m)	1.48 (m)	1.48 (m)	1.81 (m)	2.02 (m)
**16b**	2.10 (m)	1.79 (m)	2.03 (m)	1.98 (m)	2.14 (m)	2.11 (m)
**17-OH**		5.30 (s)				
**18**	2.20 (s)	1.48 (s)	2.18 (s)	2.22 (s)	2.18 (s)	2.20 (s)
**19**	1.52 (s)	0.85 (s)	1.57 (s)	1.59 (s)	1.32 (s)	1.54 (s)
**20**	5.28 (br q, 6.0 Hz)	4.61 (br q, 6.0 Hz)	5.11 (br q, 6.1 Hz)	4.09 (br q, 6.4 Hz)	5.28 (m)	5.28 (br q, 6.2Hz)
**21**	1.54 (d, 6.0 Hz)	1.21 (d, 6.0 Hz)	1.48 (d, 6.1 Hz)	1.36 (d, 6.4 Hz)	1.57 (d, 5.8 Hz)	1.56 (d, 6.2 Hz)
**Cinnamoyl moiety**						
**2'**	6.51 (d, 16 Hz)	5.96 (d, 16 Hz)	6.74 (d, 16 Hz)	6.97 (d, 16 Hz)	6.50 (d, 16 Hz)	6.51 (d, 16 Hz)
**3'**	7.80 (d, 16 Hz)	7.30 (d, 16 Hz)	7.93 (d, 16 Hz)	8.14 (d, 16 Hz)	7.87 (d, 16 Hz)	7.80 (d, 16 Hz)
**5', 9'**	7.35 (m)	7.27 (m)	7.66 (m)	7.52 (m)	7.35 (m)	7.37 (m)
**6', 8'**	7.33 (m)	7.10 (m)	7.41 (m)	7.32 (m)	7.33 (m)	7.31 (m)
**7'**	7.35 (m)	7.27 (m)	7.40 (m)	7.33 (m)	7.35 (m)	7.37 (m)
**(*E*)-2-Methyl-2-butenoyl or benzoyl moiety**						
**3''**	8.21 (d, 7.6 Hz)	7.90 (d, 7.2 Hz)	7.00 (d, 7.3 Hz)		8.23 (d, 7.2 Hz)	8.21 (d, 7.2 Hz)
**4''**	7.30 (m)	7.35 (m)	1.51 (d, 7.3 Hz)		7.39 (m)	7.32 (m)
**5''**	7.50 (m)	7.60 (m)	1.78 (s)		7.56 (m)	7.53 (m)
**6''**	7.30 (m)	7.35 (m)			7.39 (m)	7.32 (m)
**7''**	8.21 (d, 7.6 Hz)	7.90 (d, 7.2 Hz)			8.23 (d, 7.2 Hz)	8.21 (d, 7.2 Hz)

^(a)^ Measured at 500 MHz; ^(b)^ DMSO-*d*_6_ as solvent.

**Table 2:** ^13^C-NMR (100 MHz) of the aglycones of compounds **1**–**5** (in pyridine*-d*_5_).

NO.	δ_C_					
	1 ^(a)^	1 ^(a),(b)^	2	3	4	5 ^(a)^
**1**	27.6 (t)	26.6 (t)	27.5 (t)	27.6 (t)	38.7 (t)	27.6 (t)
**2**	26.5 (t)	25.2 (t)	26.5 (t)	26.5 (t)	29.8 (t)	26.5 (t)
**3**	74.9 (d)	73.3 (d)	74.9 (d)	74.9 (d)	77.5 (d)	74.9 (d)
**4**	39.0 (t)	37.4 (t)	39.0 (t)	39.0 (t)	39.1 (t)	39.1 (t)
**5**	74.7 (s)	72.6 (s)	74.7 (s)	74.8 (s)	139.1 (s)	74.7 (s)
**6**	136.7 (d)	135.4 (d)	136.6 (d)	136.1 (d)	119.3 (d)	136.7 (d)
**7**	127.3 (d)	124.0 (d)	127.3 (d)	127.6 (d)	34.8 (t)	127.3 (d)
**8**	74.0 (s)	72.6 (s)	74.0 (s)	73.8 (s)	74.2 (s)	74.0 (s)
**9**	36.6 (d)	34.9 (d)	36.5 (d)	36.6 (d)	44.0 (d)	36.6 (d)
**10**	39.6 (s)	38.3 (s)	39.6 (s)	39.6 (s)	37.2 (s)	39.6 (s)
**11**	23.6 (t)	22.3 (t)	23.7 (t)	23.6 (t)	25.6 (t)	23.6 (t)
**12**	75.8 (d)	74.2 (d)	75.6 (d)	75.9 (d)	74.6 (d)	75.8 (d)
**13**	58.1 (s)	56.8 (s)	57.9 (s)	58.1 (s)	57.0 (s)	58.1 (s)
**14**	88.2 (s)	87.1 (s)	88.1 (s)	88.9 (s)	88.9 (s)	88.2 (s)
**15**	33.1 (t)	32.1 (t)	33.1 (t)	33.3 (t)	33.7 (t)	33.1 (t)
**16**	34.3 (t)	33.5 (t)	34.2 (t)	33.5 (t)	34.0 (t)	34.3 (t)
**17**	87.7 (s)	86.5 (s)	87.7 (s)	87.9 (s)	87.5 (s)	87.7 (s)
**18**	12.4 (q)	11.6 (q)	12.3 (q)	12.7 (q)	11.5 (q)	12.5 (q)
**19**	21.5 (q)	20.9 (q)	21.5 (q)	21.6 (q)	17.9 (q)	21.6 (q)
**20**	75.3 (d)	73.9 (d)	74.4 (d)	70.4 (d)	75.8 (d)	75.3 (d)
**21**	15.6 (q)	15.0 (q)	15.5 (q)	19.6 (q)	15.3 (q)	15.6 (q)
**Cinnamoyl moiety**						
**1'**	166.8 (s)	165.5 (s)	166.7 (s)	167.0 (s)	166.8 (s)	166.8 (s)
**2'**	120.1 (d)	118.9 (d)	120.3 (d)	119.6 (d)	120.3 (d)	120.2 (d)
**3'**	143.9 (d)	143.2 (d)	143.7 (d)	145.2 (d)	143.8 (d)	143.9 (d)
**4'**	134.8 (s)	133.9 (s)	134.8 (s)	134.9 (s)	134.9 (s)	134.8 (s)
**5', 9'**	128.5 (d)	128.0 (d)	128.5 (d)	128.6 (d)	128.5 (d)	128.5 (d)
**6', 8'**	129.1 (d)	128.8 (d)	129.2 (d)	129.2 (d)	129.1 (d)	129.1 (d)
**7'**	130.4 (d)	130.1 (d)	130.5 (d)	130.5 (d)	130.4 (d)	130.4 (d)
**(*E*)-2-Methyl-2-butenoyl or benzoyl moiety**						
**1''**	165.6 (s)	164.6 (s)	166.7 (s)		165.6 (s)	165.6 (s)
**2''**	131.2 (d)	130.3 (d)	129.4 (s)		131.2 (d)	131.2 (d)
**3''**	130.2 (d)	129.4 (d)	137.7 (d)		130.2 (d)	130.2 (d)
**4''**	128.7 (d)	128.5 (d)	14.1 (q)		128.7 (d)	128.7 (d)
**5''**	133.2 (d)	133.1 (d)	12.2 (q)		133.2 (d)	133.2 (d)
**6''**	128.7 (d)	128.5 (d)			128.7 (d)	128.7 (d)
**7''**	130.2 (d)	129.4 (d)			130.2 (d)	130.2 (d)

^(a)^ Measured at 125 MHz; ^(b)^ DMSO-*d*_6_ as solvent.

The ^13^C-NMR spectrum gave signals of an (*E*)-cinnamoyl at δ_C_ 166.8 (C-1'), 120.1 (C-2'), 143.9 (C-3'), 134.8 (C-4') 128.5 (C-5',9'), 129.1 (C-6',8') and 130.4 (C-7'), and a benzoyl group at δ_C_ 165.6 (C-1''). 131.2 (C-2''), 130.2 (C-3'',7''), 128.7 (C-4'',6'') and 133.2 (C-5''). The locations of the cinnamoyl on C-12 and the benzoyl on C-20 were confirmed by the HMBC experiment which was demonstrated by correlations from δ_H_ 5.38 (H-12) to C-1' (δ_C_ 166.8) and δ_H_ 5.28 (H-20) to C-1'' (δ_C_ 165.6). Based on above evidence, the aglycone part of compound **1** was determined to be 12-*O*-(*E*)-cinnamoyl-20-*O*-benzoyl-(20*S*)-pregn-6-ene-3β,5α,8β,12β,14β,17β,20-heptol.

The ^1^H-NMR spectrum (Table 3) of **1** displayed five anomeric proton signals at δ_H_ 5.17 (brd, *J* = 10.9 Hz, 1H), 4.68 (brd, *J* = 9.6 Hz, 1H), 4.88 (d, *J* = 7.8 Hz, 1H), 5.09 (d, *J* = 7.8 Hz, 1H) and 5.20 (d, *J* = 7.8 Hz, 1H), indicating the existence of five β-configurated glycosyl linkages. The methyl signals at δ_H_ 1.39 (d, *J* = 6.0 Hz, 3H), 1.64 (d, *J* = 5.2 Hz, 3H) and 1.75 (d, *J* = 5.6 Hz, 3H) and the COSY data suggested the presence of three 6-deoxysugars. Since some of the sugar signals were seriously overlapped, compound **1** was subjected to enzymatic hydrolysis catalyzed by β-glucosidase, which afforded compound **5**.

Compound **5** was a colorless powder. Its HRESIMS spectrum showed a pseudo-molecular ion peak at *m/z* 1,260.6208 [M+NH_4_]^+^ (calcd. 1,260.6166), corresponding to a molecular weight of 1242, 162 mass number less than that of compound **1**. The ^1^H- and ^13^C-NMR data of **5** were nearly identical to that of **1** except the loss of the anomeric proton at δ_H_ 5.20 (1H, d, *J* = 7.8 Hz) and a set of oxymethine signals, which were assignable to a terminal glucose group.

**Figure 2:**
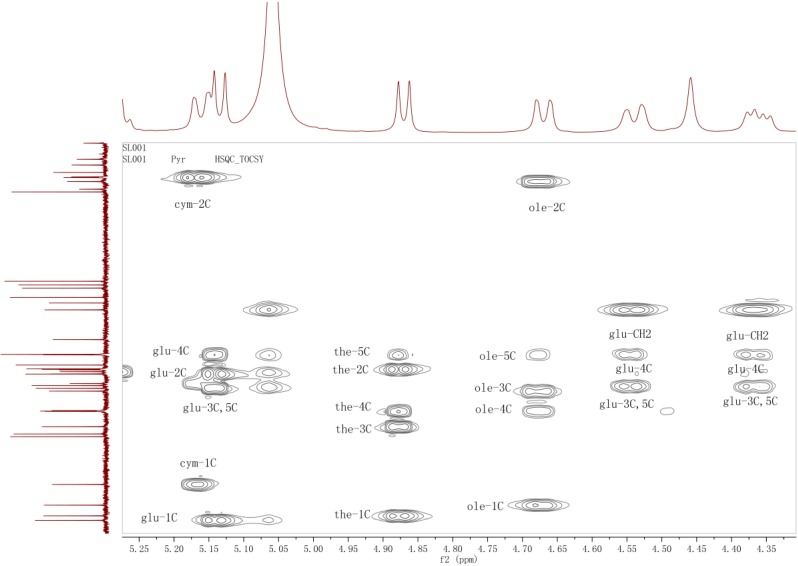
The HMQC-TOCSY spectrum of the glycosyl part of **5**.

Starting from the anomeric protons at δ_H_ 5.17, 4.68, 4.88 and 5.14, the signals of four sugar fragments (S_1_–S_4_) in the glycosyl moiety of **5** were assigned by analysis of the HMQC-TOCSY spectrum (Figure 2) combined with the HMQC and COSY data.

**Figure 3:**
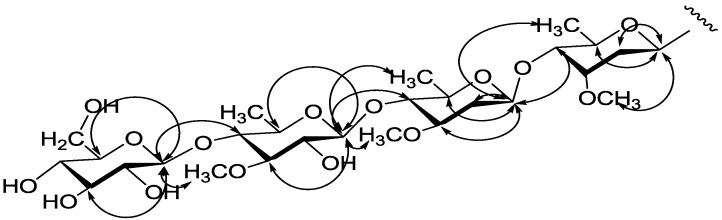
Key NOE of the sugars of **5**.

The glycosyl moiety was found being composed of a β-linked terminal glucose and three β-linked deoxy-sugars (see Table 3 and Table 4). In the HMBC spectrum, three methoxy groups (δ_H_ 3.52, S_1_-OCH_3_; 3.51, S_2_-OCH_3_ and 3.94, S_3_-OCH_3_) were correlated with the carbon signals at δ_C_ 77.7 (S_1_-C-3), 79.1 (S_2_-C-3) and 86.2 (S_3_-C-3), indicating that each of the deoxy-sugars bore a methoxy group on C-3. Identification of the deoxy-sugars was reached by inspection of the NOE spectra (Figure 3). Nuclear Overhauser Effects were observed between the anomeric proton at δ_H_ 5.17 (S_1_-H-1) and the signals at δ_H_ 4.16 (S_1_-H-5), 2.23 (S_1_-H-2) and 3.52 (S_1_-OCH_3_), suggesting that S_1_ is a β-cymaropyranose. Similarly S_2_ and S_3_ were identified as β-oleandropyranose and β-thevetopyranose. As regards the configuration of the deoxy-sugars, previous studies revealed that all the β-linked 2-deoxysugars have the D-configuration, whereas the α-linked sugars are mostly l-sugars [34]. Further, chemical shift values for C-2 of the 2-deoxysugars (cymarose, oleandrose and digitoxose) can be used as argument to determine its configuration [35]. The chemical shift of C-2 in the l-sugars is less than 35.0 ppm, but that of C-2 in the D-sugars appears above 36.0 ppm [36,37,38,39]. In the case of compound **5**, the two 2-deoxysugars were β-linked and their C-2 signals occurred at δ_C_ 36.7 and 37.5, respectively. The cymarose and oleandrose moieties had thus D-configuration. Configuration of the β-thevetopyranosyl unit was presumed to be D-form because its NMR data were close to those in the literature [40,41,42] and l-thevetopyranose [43] is rarely found in Nature.

**Figure 4:**
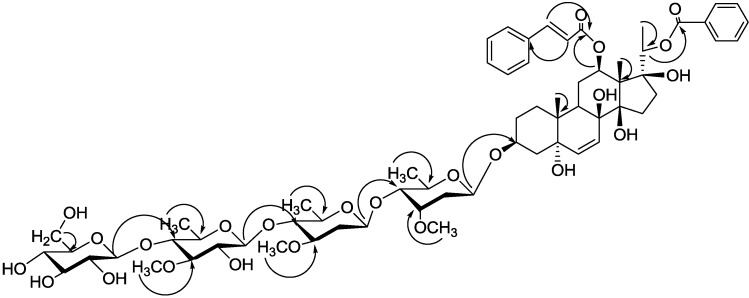
Key HMBC correlations of **5**.

The sequential lingkage of the four sugars shown in Figure 3 was deduced from the NOE correlations between S_2_-H-1/S_1_-H-4, S_3_-H-1/S_2_-H-4 and S_4_-H-1/S_3_-H-4 and the HMBC (Figure 4) cross peaks between S_2_-H-1/S_1_-C-4 (δ_C_ 83.0), S_3_-H-1/S_2_-C-4 (δ_C_ 83.1) and S_4_-H-1/S_3_-C-4 (δ_C_ 83.2). This was confirmed by comparison of the NMR data of the sugar moiety with that of verticilloside A [44], which carried the same glycosyl moiety. Based on above evidences, the structure of compound **5** is elucidated as 12-*O*-(*E*)-cinnamoyl-20-*O*-benzoyl-(20*S*)-pregn-6-ene-3β,5α,8β,12β,14β,17β,20-heptol, 3-*O*-β-d-glucopyranosyl-(1→4)-β-d-thevetopyranosyl-(1→4)-β-d-oleandropyranosyl-(1→4)-β-d-cymaropyranoside, named gymsylvestroside E.

**Table 3:** ^1^H-NMR (400 MHz) of the glycosyl part of compounds **1**–**5** (in pyridine*-d*_5_, *J* in Hz).

Sugar	NO.	δ_H_ (*J* in Hz)				
		1 ^(a)^	2	3	4	5 ^(a)^
Cym	**1**	5.17 (brd, 10.9 Hz)	5.17 (brd, 10.9 Hz)	5.17 (brd, 10.9 Hz)	5.29 (brd, 10.9 Hz)	5.17 (brd, 10.5 Hz)
	**2a**	1.73 (m)	1.73 (m)	1.73 (m)	1.89 (m)	1.73 (m)
	**2b**	2.20 (m)	2.20 (m)	2.23 (m)	2.31 (m)	2.23 (m)
	**3**	3.98 (m)	3.98 (m)	3.98 (m)	3.98 (m)	3.98 (m)
	**4**	3.43 (m)	3.43 (m)	3.43 (m)	3.43 (m)	3.42 (m)
	**5**	4.16 (m)	4.16 (m)	4.16 (m)	4.16 (m)	4.16 (m)
	**6**	1.39 (d, 6.0 Hz)	1.39 (d, 6.0 Hz)	1.39 (d, 6.0 Hz)	1.46 (d, 6.0 Hz)	1.39 (d, 6.0 Hz)
	**OMe**	3.52 (s)	3.52 (s)	3.52 (s)	3.59 (s)	3.52 (s)
Ole	**1**	4.68 (brd, 9.6 Hz)	4.68 (brd, 9.6 Hz)	4.68 (brd, 9.6 Hz)	4.70 (brd, 9.6 Hz)	4.68 (brd, 9.6 Hz)
	**2a**	2.47 (m)	2.47 (m)	2.46 (m)	2.49 (m)	2.48 (m)
	**2b**	1.73 (m)	1.73 (m)	1.73 (m)	1.76 (m)	1.73 (m)
	**3**	3.57 (m)	3.57 (m)	3.57 (m)	3.57 (m)	3.57 (m)
	**4**	3.59 (m)	3.59 (m)	3.59 (m)	3.59 (m)	3.52 (m)
	**5**	3.55 (m)	3.58 (m)	3.55 (m)	3.51 (m)	3.51 (m)
	**6**	1.64 (d, 5.2 Hz)	1.64 (d, 5.2 Hz)	1.64 (d, 5.2 Hz)	1.68 (d, 5.2 Hz)	1.64 (d, 5.2 Hz)
	**OMe**	3.50 (s)	3.51 (s)	3.51 (s)	3.51 (s)	3.51 (s)
The	**1**	4.88 (d, 7.8 Hz)	4.88 (d, 7.6 Hz)	4.88 (d, 7.6 Hz)	4.88 (d, 7.6 Hz)	4.88 (d, 7.6 Hz)
	**2**	3.90 (m)	3.90 (m)	3.90 (m)	3.90 (m)	3.90 (m)
	**3**	3.67 (m)	3.68 (m)	3.67 (m)	3.67 (m)	3.69 (m)
	**4**	3.83 (m)	3.83 (m)	3.83 (m)	3.83 (m)	3.88 (m)
	**5**	3.75 (m)	3.77 (m)	3.75 (m)	3.75 (m)	3.75 (m)
	**6**	1.75 (d, 5.6 Hz)	1.76 (d, 5.6 Hz)	1.75 (d, 5.6 Hz)	1.75 (d, 5.6 Hz)	1.75 (d, 5.6 Hz)
	**OMe**	3.91 (s)	3.82 (s)	3.82 (s)	3.91 (s)	3.94 (s)
Glc	**1**	5.09 (d, 7.8 Hz)	5.09 (d, 7.8 Hz)	5.09 (d, 7.8 Hz)	5.09 (d, 7.8 Hz)	5.14 (d, 7.8 Hz)
	**2**	4.02 (m)	4.02 (m)	4.02 (m)	4.02 (m)	4.04 (m)
	**3**	4.28 (m)	4.28 (m)	4.28 (m)	4.28 (m)	4.25 (m)
	**4**	4.31 (m)	4.31 (m)	4.30 (m)	4.31 (m)	4.23 (m)
	**5**	3.93 (m)	3.93 (m)	3.93 (m)	3.93 (m)	3.98 (m)
	**6a**	4.30 (m)	4.30 (m)	4.30 (m)	4.30 (m)	4.32 (m)
	**6b**	4.50 (m)	4.50 (m)	4.50 (m)	4.50 (m)	4.54 (m)
Glc	**1**	5.20 (d, 7.8 Hz)	5.20 (d, 8.5 Hz)	5.20 (d, 8.5 Hz)	5.20 (d, 8.5 Hz)	
	**2**	4.10 (m)	4.10 (m)	4.10 (m)	4.10 (m)	
	**3**	4.23 (m)	4.23 (m)	4.23 (m)	4.23 (m)	
	**4**	4.19 (m)	4.16 (m)	4.19 (m)	4.19 (m)	
	**5**	4.04 (m)	4.04 (m)	4.04 (m)	4.04 (m)	
	**6a**	4.30 (m)	4.32 (m)	4.30 (m)	4.31 (m)	
	**6b**	4.53 (m)	4.53 (m)	4.53 (m)	4.53 (m)	

^(a)^ Measured at 500 MHz; Cym: β-d-cymaropyranosyl, Ole: β-d-oleandropyranosyl, The: β-d-thevetopyranosyl, Glc: β-d-glucopyranosyl.

**Table 4:** ^13^C (100 MHz) NMR of the glycosyl part of compounds **1**–**5** (in pyridine*-d*_5_).

Sugar	NO.		δ_C_				
			1 ^(a)^	2	3	4	5 ^(a)^
Cym		**1**	97.7 (d)	97.6 (d)	97.7 (d)	96.3 (d)	97.7 (d)
		**2**	36.7 (t)	36.7 (t)	36.7 (t)	37.2 (t)	36.7 (t)
		**3**	77.7 (d)	77.6 (d)	77.7 (d)	77.8 (d)	77.7 (d)
		**4**	83.0 (d)	83.0 (d)	83.0 (d)	83.2 (d)	83.0 (d)
		**5**	68.9 (d)	68.9 (d)	68.9 (d)	68.8 (d)	68.9 (d)
		**6**	18.5 (q)	18.4 (q)	18.5 (q)	18.4 (q)	18.5 (q)
		**OMe**	58.7 (q)	58.6 (q)	58.7 (q)	58.8 (q)	58.7 (q)
Ole		**1**	101.8 (d)	101.8 (d)	101.8 (d)	101.8 (d)	101.8 (d)
		**2**	37.5 (t)	37.5 (t)	37.5 (t)	37.6 (t)	37.5 (t)
		**3**	79.1 (d)	79.1 (d)	79.2 (d)	79.2 (d)	79.1 (d)
		**4**	83.2 (d)	83.2 (d)	83.2 (d)	83.4 (d)	83.1 (d)
		**5**	71.9 (d)	71.8 (d)	71.8 (d)	71.8 (d)	71.9 (d)
		**6**	18.6 (q)	18.6 (q)	18.6 (q)	18.6 (q)	18.6 (q)
		**OMe**	57.3 (q)	57.3 (q)	57.3 (q)	57.3 (q)	57.4 (q)
The		**1**	103.9 (d)	104.0 (d)	103.9 (d)	103.9 (d)	104.0 (d)
		**2**	74.9 (d)	74.9 (d)	74.8 (d)	74.9 (d)	74.8 (d)
		**3**	86.3 (d)	86.3 (d)	86.3 (d)	86.3 (d)	86.2 (d)
		**4**	83.3 (d)	83.3 (d)	83.4 (d)	83.4 (d)	83.2 (d)
		**5**	71.9 (d)	71.8 (d)	71.9 (d)	71.9 (d)	71.9 (d)
		**6**	18.7 (q)	18.7 (q)	18.7 (q)	18.7 (q)	18.7 (q)
		**OMe**	60.6 (q)	60.6 (q)	60.6 (q)	60.6 (q)	60.6 (q)
Glc		**1**	104.5 (d)	104.5 (d)	104.6 (d)	104.6 (d)	104.8 (d)
		**2**	75.3 (d)	75.3 (d)	75.3 (d)	75.3 (d)	75.8 (d)
		**3**	76.8 (d)	76.8 (d)	76.8 (d)	76.8 (d)	78.6 (d)
		**4**	81.5 (d)	81.5 (d)	81.5 (d)	81.5 (d)	71.9 (d)
		**5**	76.2 (d)	76.2 (d)	76.2 (d)	76.2 (d)	78.1 (d)
		**6**	62.3 (t)	62.2 (t)	62.3 (t)	62.3 (t)	63.0 (t)
Glc		**1**	104.9 (d)	104.9 (d)	104.9 (d)	104.9 (d)	
		**2**	74.7 (d)	74.7 (d)	74.9 (d)	74.7 (d)	
		**3**	78.2 (d)	78.2 (d)	78.2 (d)	78.2 (d)	
		**4**	71.5 (d)	71.5 (d)	71.5 (d)	71.4 (d)	
		**5**	78.4 (d)	78.4 (d)	78.4 (d)	78.4 (d)	
		**6**	62.4 (t)	62.3 (t)	62.4 (t)	62.4 (t)	

^(a)^ Measured at 125 MHz; Cym: β-d-cymaropyranosyl, Ole: β-d-oleandropyranosyl, The: β-d-thevetopyranosyl, Glc: β-d-glucopyranosyl.

As mentioned above, compound **5** was derived from **1** by removing a terminal glucose. In the HMBC spectrum of **1**, the anomeric proton (δ_H_ 5.20, d, *J* = 7.8 Hz, 1H) of the terminal glucose was correlated with S_4_-C-4 (δ_C_ 81.5). The signals of S_4_-C-3, S_4_-C-4 and S_4_-C-5 exhibited glycosylation shifts of Δ −1.8 ppm, +9.6 ppm and −1.9 ppm, respectively. The terminal glucose was therefore determined to locate on C-4 of another glucose moiety. These evidences allowed to elucidate the structure of compound **1** as 12-*O*-(*E*)-cinnamoyl-20-*O*-benzoyl-(20*S*)-pregn-6-ene-3β,5α,8β,12β,14β,17β,20-heptol, 3-*O*-β-d-glucopyranosyl-(1→4)-β-d-glucopyranosyl-(1→4)-β-d-thevetopyranosyl-(1→4)-β-d-oleandropyranosyl-(1→4)-β-d-cymaropyranoside, named gymsylvestroside A.

Compound **2** was isolated as a colorless powder. Its molecular formula was determined to be C_68_H_102_O_29_ by HRESIMS *m/z* 1,383.6593 [M+H]^+^ (calcd. 1,383.6585). Comparing to the ^13^C-NMR data of **1**, the benzoyl signals at δ_C_ 133.2, 128.7 (overlapped), 130.2 (overlapped), 131.2 and 165.6 disappeared in the spectrum of **2**, while signals for another acyl group were observed at δ_C_ 12.2, 14.1, 129.4, 137.7 and 166.7. This group was identified as 2-methyl-2-butenoyl by inspection of the ^1^H,^1^H-COSY, HMQC and HMBC spectra and comparison with reported data [31,32,33]. The HMBC coupling between H-20 (δ_H_ 5.11) and the carbonyl carbon (δ_C_ 166.7) of the 2-methyl-2-butenoyl group confirmed the connection of this group to C-20. The ^1^H-NMR showed five anomeric proton signals at δ_H_ 5.17 (brd, *J* = 10.9 Hz), 4.68 (brd, *J* = 9.6 Hz), 4.88 (d, *J* = 7.8 Hz), 5.09 (d, *J* = 7.8 Hz) and 5.20 (d, *J* = 7.8 Hz). The nearly identical NMR data of the sugars and the HMBC correlations between δ_H_ 5.17/δ_C_ 74.9 (C-3), δ_H_ 4.68/δ_C_ 83.0 (cym-C-4), δ_H_ 4.88/δ_C_ 83.2 (ole-C-4), δ_H_ 5.09/δ_C_ 83.3 (the-C-4) and δ_H_ 5.20/δ_C_ 81.5 (glu-C-4) indicated that compound **2** carried the same sugar moiety as that of **1**. The structure of **2** was thus established as 12-*O*-(*E*)-cinnamoyl-(20*S*)-*O*-(*E*)-2-methyl-2-butenoyl-(20*S*)-pregn-6-ene-3β,5α,8β,12β,14β,17β,20-heptol, 3-*O*-β-d-glucopyranosyl-(1→4)-β-d-glucopyranosyl-(1→4)-β-d-thevetopyranosyl-(1→4)-β-d-oleandropyranosyl-(1→4)-β-d-cymaropyranoside, named gymsylvestroside B.

Compound **3**, a colorless powder, was designated a molecular formula of C_63_H_96_O_28,_ based on the pseudo-molecular ion peak at *m/z* 1,323.5978 [M+Na]^+^ (calcd. 1,323.5986) in its HRESIMS spectrum. The NMR data of **3** were almost identical to those of **1** (Table 1, Table 2, Table 3 and Table 4), except for the loss of the signals of the benzoyl group. Comparing to the data of **1**, the C-21 signal (δ_C_ 19.6) in the spectrum of **3** moved 3 ppm downfield and the C-20 (δ_C_ 70.4) shifted 4.9 ppm upfield, showing the presence of a free hydroxyl group on C-20. Further analysis of the 2D NMR data elucidated the structure of compound **3** as 12-*O*-(*E*)-cinnamoyl-(20*S*)-pregn-6-ene-3β,5α,8β,12β,14β,17β,20-heptol, 3-*O*-β-d-glucopyranosyl-(1→4)-β-d-glucopyranosyl-(1→4)-β-d-thevetopyranosyl-(1→4)-β-d-oleandropyranosyl-(1→4)-β-d-cymaropyranoside, named gymsylvestroside C.

Compound **4** was obtained as a colorless powder. Its HRESIMS data (*m/z* 1,411.6318 [M+Na]^+^, calcd. 1411.6299) suggested a molecular formula of C_70_H_100_O_28_, which has one oxygen less than that of compound **1**. The ^1^H- and ^13^C-NMR data of **4** were only slightly different from that of **1**. The C-5 signal (δ_C_ 74.7) of **1** disappeared in the ^13^C-NMR spectrum of **4**, while an additional methene signal occured at δ_C_ 34.8 (C-7). The signals of the double bond in the pregnane skeleton shifted to δ_C_ 119.3 and 139.1. The ^1^H-NMR of **4** displayed an olefinic proton at δ_H_ 5.36 (H-6), which correlated in the HMBC spectrum with the methyl carbon at δ_C_ 37.2 (C-10). The signal of Me-19 (δ_H_ 1.32) showed HMBC correlation with a disubstituted vinyl carbon at δ_C_ 139.1. These facts concluded the presence of a double bond between C-5 and C-6. Comparing to the reported data for gymnpregoside F [33], the pregnane skeleton of **4** was determined to be (20*S*)-pregn-5-ene-3β,17β,14β,12β,5α,8β,20-heptol. Detailed investigation of the 1D and 2D NMR spectra of **4** revealed the presence of signals of a cinnamoyl, a benzoyl group and the same sugar moiety as in **1**. The location of the cinnamoyl at C-12, the benzoyl at C-20 and the sugar moiety at C-3 was confirmed by the HMBC correlations from δ_H_ 5.25 (H-12) to δ_C_ 166.8, δ_H_ 5.28 (H-20) to δ_C_ 165.6 and δ_H_ 5.29 (S1-H-1) to δ_C_ 77.5. The structure of **4** could thus be determined as 12-*O*-(*E*)-cinnamoyl-20-*O*-benzoyl-(20*S*)-pregn-5-ene-3β,17β,14β,12β,5α,8β,20-heptol, 3-*O*-β-d-glucopyranosyl-(1→4)-β-d-glucopyranosyl-(1→4)-β-d-thevetopyranosyl-(1→4)-β-d-oleandropyranosyl-(1→4)-β-d-cymaropyranoside, named gymsylvestroside D.

### 2.2. Biological Activity Assay

The *Saccharomces cerevisiae* α-glucosidase inhibition activities of compounds **1**–**4** were assayed using *p*-nitrophenyl-α-d-glucopyranoside (*p*NPG) as substrate and acarbose (J&K) as positive control. Compounds **1**–**4** did not show significant inhibitory effect (Table 5).

**Table 5:** The inhibitory activity of the compounds **1**–**4**.

Sample	Concentration	OD	Inhibition Ratio
	(mg/mL)	Average ± RSD	(%)
Negetive control	0	1.8573 ± 0.0129	
Compound **1**	1.02	1.7661 ± 0.0077	4.9
Compound **2**	1.07	1.7860 ± 0.0033	3.8
Compound **3**	1.11	1.7356 ± 0.0025	6.6
Compound **4**	1.16	1.7901 ± 0.0029	3.6
Acarbose	0.50	1.3654 ± 0.0026	26

## 3. Experimental

### 3.1. General Procedures

Optical rotations were measured on an Optical Activity Limited polAAr 3005 spectropolarimeter (Optical Activity Limited, Ramsey, UK). IR and UV spectra were taken out on a Nicolet-Is5 infrared spectrometer (Thermo Fisher, Boston, MA, USA) and a Cintra-20 UV-Vis spectrometer (GBC, Melbourne, Australia), respectively. HRESIMS was obtained with Waters G2 Q-TOF (Waters, Milford, MA, USA) or Agilent 6520 Q-TOF (Agilent Technologies, Santa Clara, CA, USA) mass spectrometer. 1D and 2D NMR spectra were recorded with JNM-ECA-400 (JEOL Ltd., Tokyo, Japan) or Bruker AVANCE III 500 (Bruker Biospin, Switzerland) superconducting NMR spectrometer. TMS and pyridine-*d*_5_ were used as internal standards for ^1^H- and ^13^C-NMR measurements, respectively. Preparative HPLC was carried out with Waters Autopurification System (Waters). YMC-PACK ODS-A (S-5 um, 20 × 250 mm; YMC Co. Ltd., Kyoto, Japan) column was used in preparative HPLC. Microplate reader-MaxM5 (Molecular Devices, Santa Clara, CA, USA) was used to determine the absorbance of the enzymatic reaction. Silica gel (SiO_2_; 200–300 mesh; Qingdao Marine Chemical Inc., Qingdao, China), MCI GEL resin (50 um; Mitsubishi Plastics, Tokyo, Japan) and D-101 macroporous resin (16–60 mesh; Nankai University Chemical Plant, Tianjin, China) were used for column chromatography. Acarbose (lot No. 298087) was purchased from J&K Scientific Co. Ltd. α-Glucosidases (lot No. 1001604919) and *p*-nitrophenyl-α-d-glucopyranodase (*p*NPG) (lot No. 101381642) were purchased from Sigma Chemical Co. (St. Louis, MO, USA). β-Glucosidases (*Aspergillus niger*) was provided by Baiping Ma (Institute of Radiation Medicine, Academy for Military Medical Science). The other chemicals used in this study were of analytical grade.

### 3.2. Plant Materials

The stems of *G. sylvestre* were obtained from the Exhibition Center of Guangxi University of Chinese Medicine in August, 2010 and identified as *G. sylvestre* by Professor Wenhui Tan at Guangxi University of Chinese Medicine. A voucher specimen was preserved at the herbarium of Institute of Pharmacology and Toxicology with the reference number Gs201008002.

### 3.3. Extraction and Isolation

The dried stems (20 kg) of *G. sylvestre* were extracted with 50% ethanol three times (120 L × 1 h each). Concentration of the combined extracts under reduced pressure afforded 2 kg dry mass. The concentrate was suspended in water and extracted with *n*-butanol to give an *n*-butanol soluble extract (750 g) which was adsorbed on a macroporous resin column. The column was washed at first with water, and then eluted with 15%, 30%, 50%, 70% and 95% ethanol successively. The eluate (120 g) of 50% ethanol was fractionated on a silica gel column, eluting sequentially with chloroform/methanol (20:1-1:5, v/v) to give six fractions (A_1_-A_6_). Fraction A_4_ (6 g) was dissolved in water and adsorbed with a MCI resin using aqueous methanol (0-45%) as elute, giving fractions A_4-1_-A_4-5_. Separation of A_4-3_ (2 g) by preparative HPLC with an ODS-A column (20 × 250 mm), eluting with MeOH/H_2_O (80:20) gave compound **1** (900 mg) and **2** (100 mg). HPLC separation of A_4-4_ (500 mg) using MeOH/H_2_O (60:40) as eluent afforded compound **3** (60 mg) and **4** (80 mg). Compound **5** (70 mg) was isolated from the hydrolysate after enzymatic hydrolysis of compound **1** (200 mg). Compounds **2** and **5** were further purified by preparative HPLC eluting with MeOH/H_2_O (75:25) to give 92 mg and 63 mg purified products for each compound. Compounds **3** and **4** were processed similarly by preparative HPLC using MeOH/H_2_O (55:45) as eluent, and each afforded 48 mg and 70 mg purified products, respectively.

### 3.4. Isolated Compounds

Compound **1**: colorless powder. ${\left[\alpha \right]}_{D}^{20}$ +80.8 (c 1.09, MeOH). HRESIMS *m/z*: 1422.6627 [M+NH_4_]^+^ (1422.6694 calcd for C_70_H_104_O_29_N). UV (MeOH) λ_max_ (log ε) 205.2 (1.84), 223.2 (1.87), 278.6 (1.81) nm. IR (film) cm^−1^: 3447, 1718, 1642, 1289, 1034. ^1^H-NMR and ^13^C-NMR: See Table 1, Table 2, Table 3 and Table 4.

Compound **2**: colorless powder. [α] ${\left[\alpha \right]}_{D}^{20}$ +71.0 (c 1.44, MeOH). HRESIMS *m/z:* 1383.6593 [M+H]^+^ (1383.6585 calcd for C_68_H_103_O_29_). UV (MeOH) λ_max_ (log ε) 217.2 (1.84), 278.6 (1.77) nm. IR (film) cm^−1^: 3431, 2513, 2161, 1057. ^1^H-NMR and ^13^C-NMR: See Table 1, Table 2, Table 3 and Table 4.

Compound **3**: colorless powder. [α] ${\left[\alpha \right]}_{D}^{20}$ +35.8 (c 1.30, MeOH). HRESIMS *m/z*: 1323.5978 [M+Na]^+^ (1323.5986 calcd for C_63_H_96_O_28_Na). UV (MeOH) λ_max_ (log ε) 206.9 (1.81), 216.3 (1.82), 277.8 (1.84) nm. IR (film) cm^−1^: 3425, 3932, 2164, 1709, 1167, 1066. ^1^H-NMR and ^13^C-NMR: See Table 1, Table 2, Table 3 and Table 4.

Compound **4**: colorless powder. [α] ${\left[\alpha \right]}_{D}^{20}$ +90.8 (c 1.05, MeOH). HRESIMS *m/z*: 1411.6318 [M+Na]^+^ (1411.6299 calcd for C_70_H_100_O_28_Na). UV (MeOH) λ_max_ (log ε) 206.1 (1.86), 223.2 (1.90), 279.5 (1.86) nm. IR (film) cm^−1^: 3446, 2937, 2518, 2161, 1709, 1167, 1105. ^1^H-NMR and ^13^C-NMR: See Table 1, Table 2, Table 3 and Table 4.

Compound **5**: colorless powder. [α] ${\left[\alpha \right]}_{D}^{20}$ +76.8 (c 1.09, MeOH). HRESIMS *m/z*: 1260.6208 [M+NH_4_]^+^ (1260.6166 calcd for C_64_H_94_O_24_N). UV (MeOH) λ_max_ (log ε) 205.2 (1.84), 223.2 (1.87), 278.6 (1.81) nm. ^1^H-NMR and ^13^C-NMR: See Table 1, Table 2, Table 3 and Table 4.

### 3.5. Enzymatic Hydrolysis of Compound **1**

Compound **1** (200 mg) was dissolved in 40mL water and then diluted with 50 mL acetic acid-sodium acetate buffer (pH = 4.97). After addition of 8 mL β-glucosidase (*Aspergilus niger*, 1 mg/mL), the solution was mixed well and incubated at 50 °C in a water bath for 24 h. The hydrolysate was loaded on a reversed-phase silica gel colunm and successively washed with water and methanol. The methanol eluate was purified by preparative HPLC to give compound **5** (70 mg).

### 3.6. α-Glucosidase Inhibitory Effect

The α-glucosidase (*S. cerevisia*) inhibitory effect of compounds **1**–**4** were determined using the method adopted previously by Hou *et al.* [45] The tested compounds (8 μL) were premixed with α*-*glucosidase (0.90 unite/mL, 20 μL) on a 96 wells microplate and diluted with 112 μL phosphate buffer (pH = 6.8). The reaction mixtures were incubated at 37 °C for 15 min and then 20 μL *p*NPG (2.45 mmol/mL) was added to start the reaction. After incubation for another 15 min at 37 °C, the reaction was stopped by adding 80 μL Na_2_CO_3_ (0.199 mmol/mL). The activity was determined by measuring the release of *p*-nitrophenol at 405 nm with a microplate reader. Acarbose was used as the positive control and DMSO (dimethyl sulfoxide) as the negative control. Inhibitory rates were calculated as follows:

Inhibition ratio (%) = [(OD_negative control_ − OD_test_)/OD_negative control_] × 100%

## 4. Conclusions

In this study, four new polyoxygenated pregnane glycosides carrying a complex pentasaccharide moiety were obtained from the 50% ethanol extract of the stem of *G. sylvestre*. Their structures were elucidated by intensive spectroscopic analysis with the help of enzymatic hydrolysis of the sugar chain. α-Glycosidase inhibitory activity of these compounds has been investigaed, but the observed data were statistically not significant.

## Supplementary Materials

Supplementary materials can be accessed at: http://www.mdpi.com/1420-3049/20/02/3050/s1.
