# Supplementary material for: New Pregnane Glycosides from Gymnema sylvestre
Source: Molecules. 2015 Feb 12;20(2):3050–66. doi: 10.3390/molecules20023050 (PMC6272391; doi:10.3390/molecules20023050)
Supplement: Supplementary file 1 [file molecules-20-03050-s001.pdf]

# Supporting Information

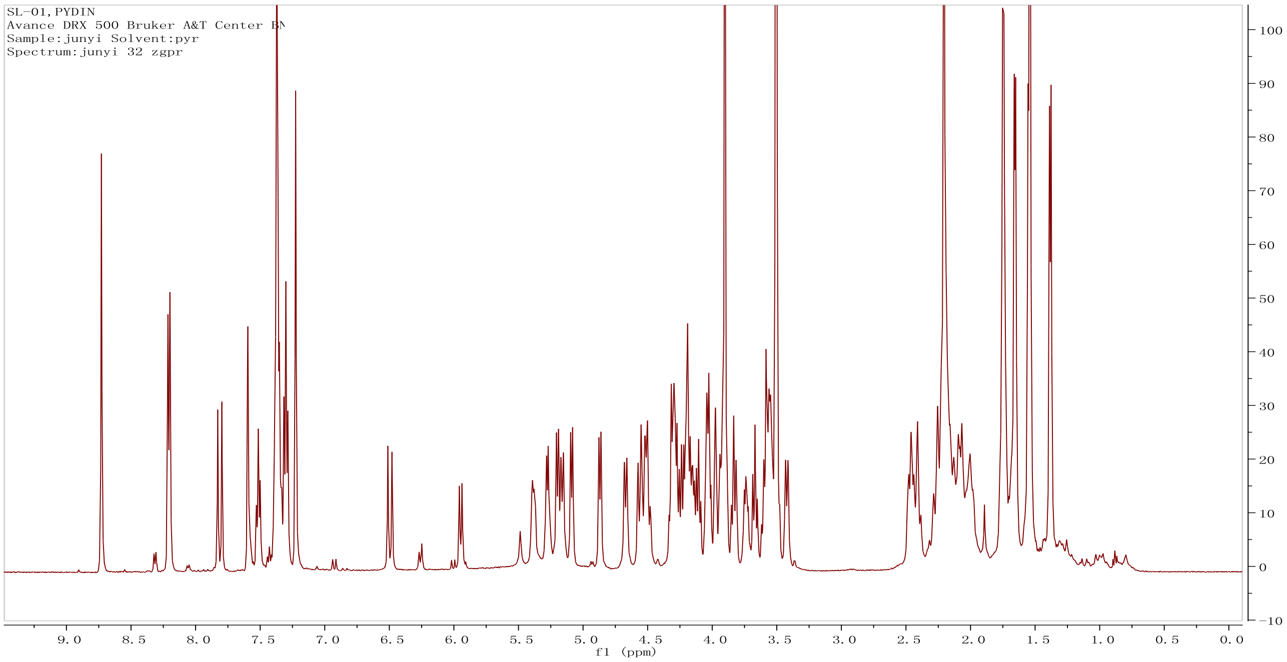


**Figure S1.** $^{1}$H-NMR (500 MHz) spectrum of **1** in Pyridine-*d*5.

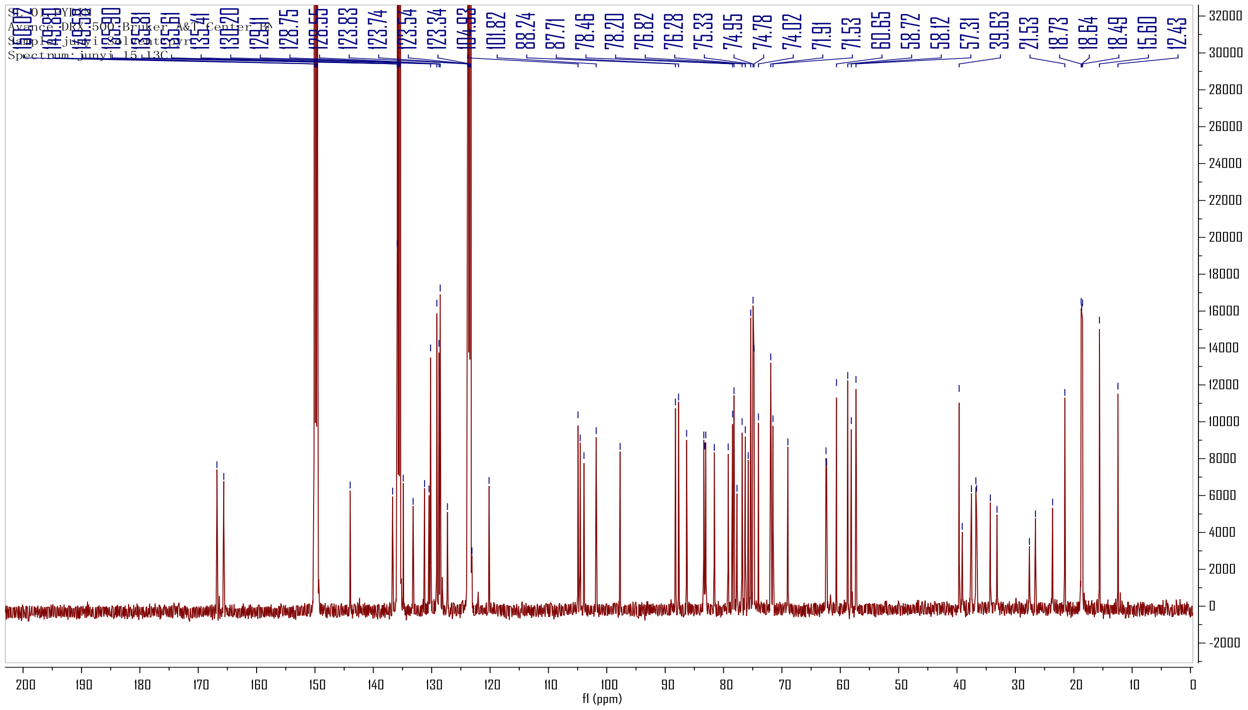


**Figure S2.** $^{13}$C-NMR (125 MHz) spectrum of **1** in Pyridine-*d*5.

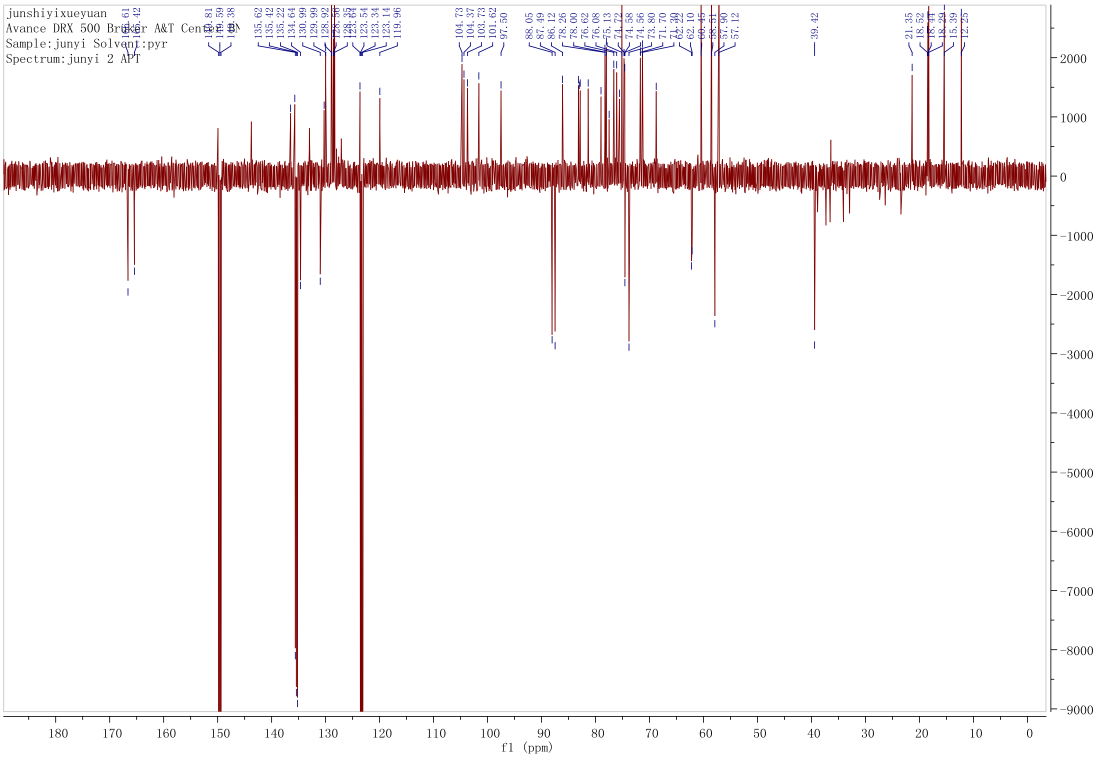


**Figure S3.** APT (500 MHz) spectrum of 1 in Pyridine-$d_5$.

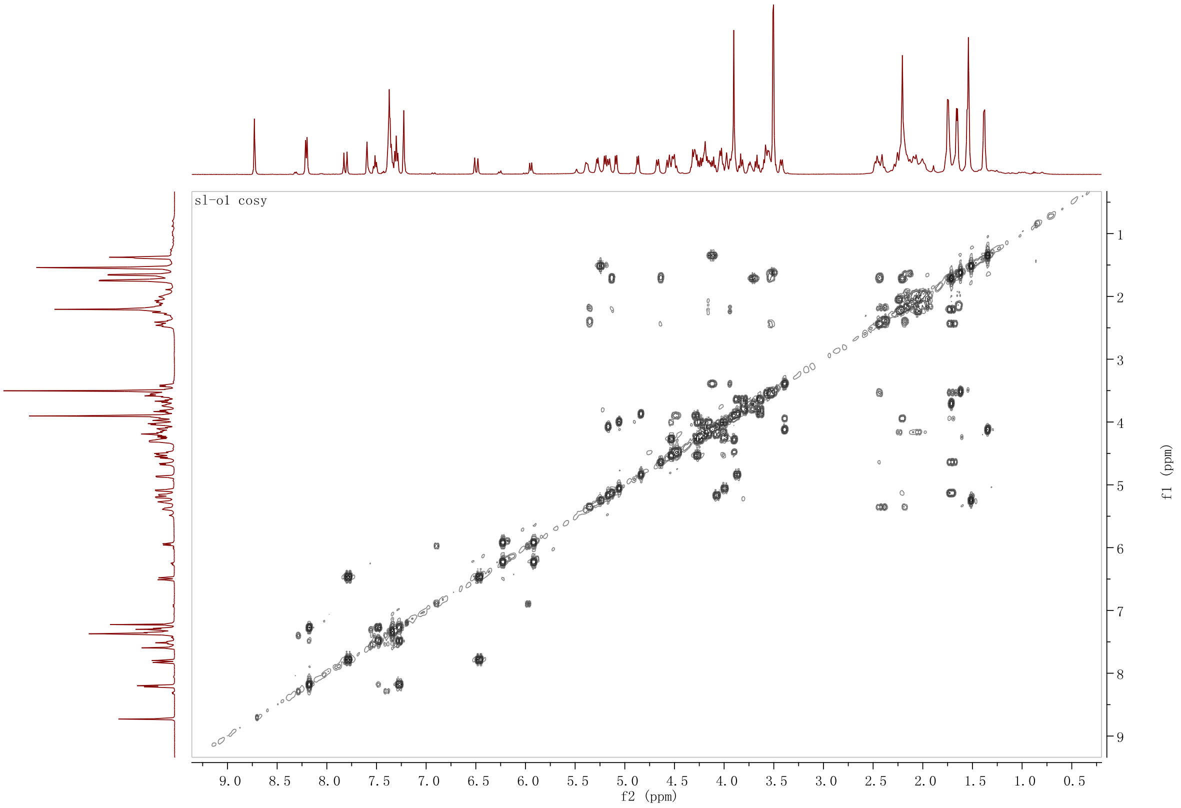


**Figure S4.** COSY (500 MHz) spectrum of **1** in Pyridine-$d_5$.

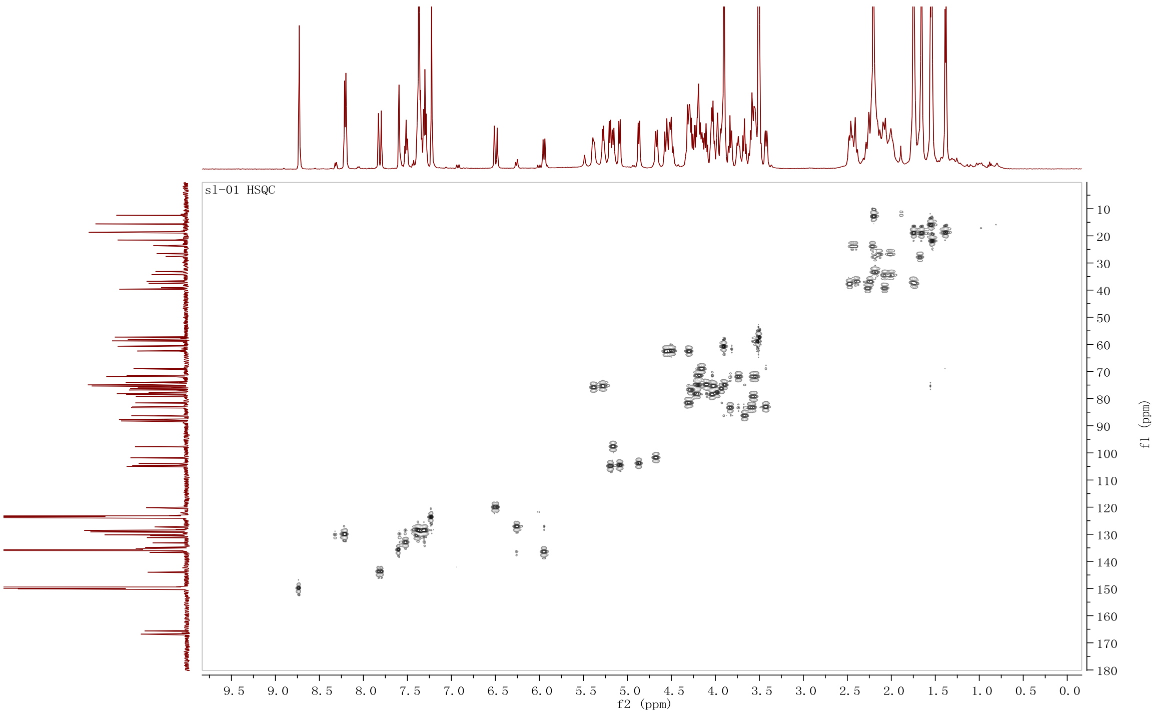


**Figure S5.** HSQC (500 MHz) spectrum of **1** in Pyridine-$d_5$.

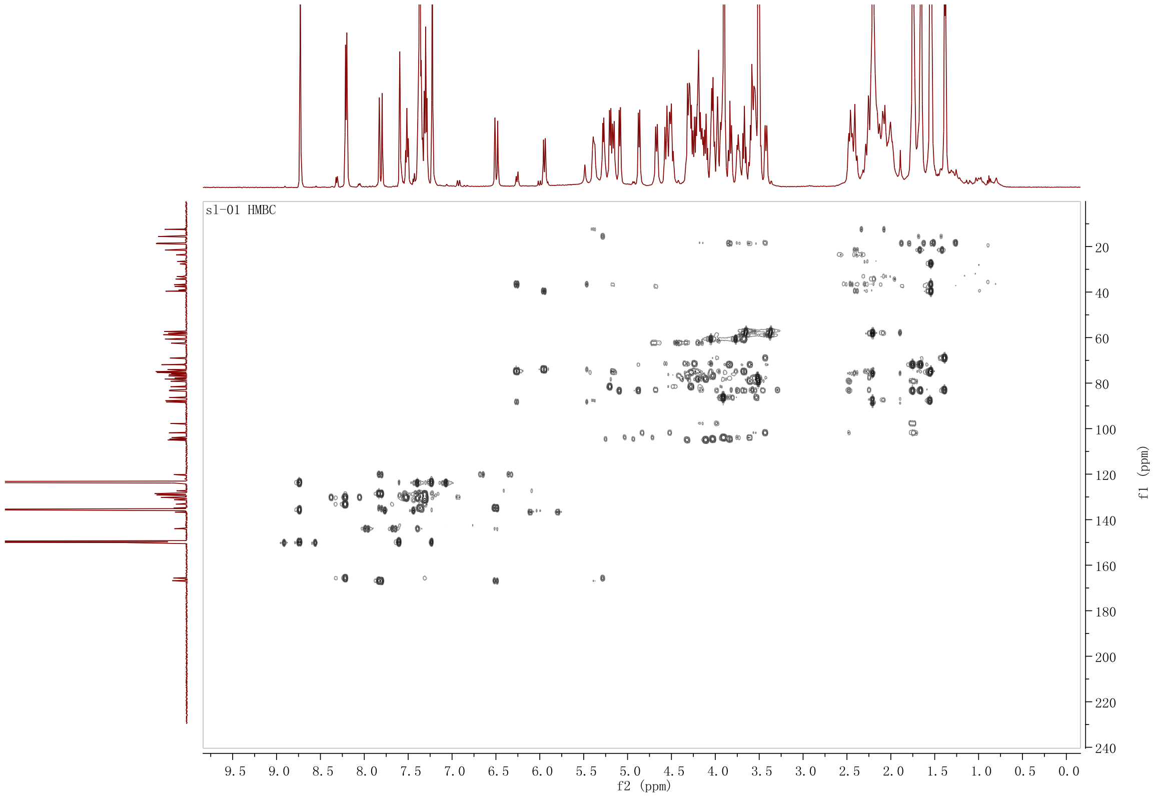


**Figure S6.** HMBC (500 MHz) spectrum of **1** in Pyridine-$d_5$.

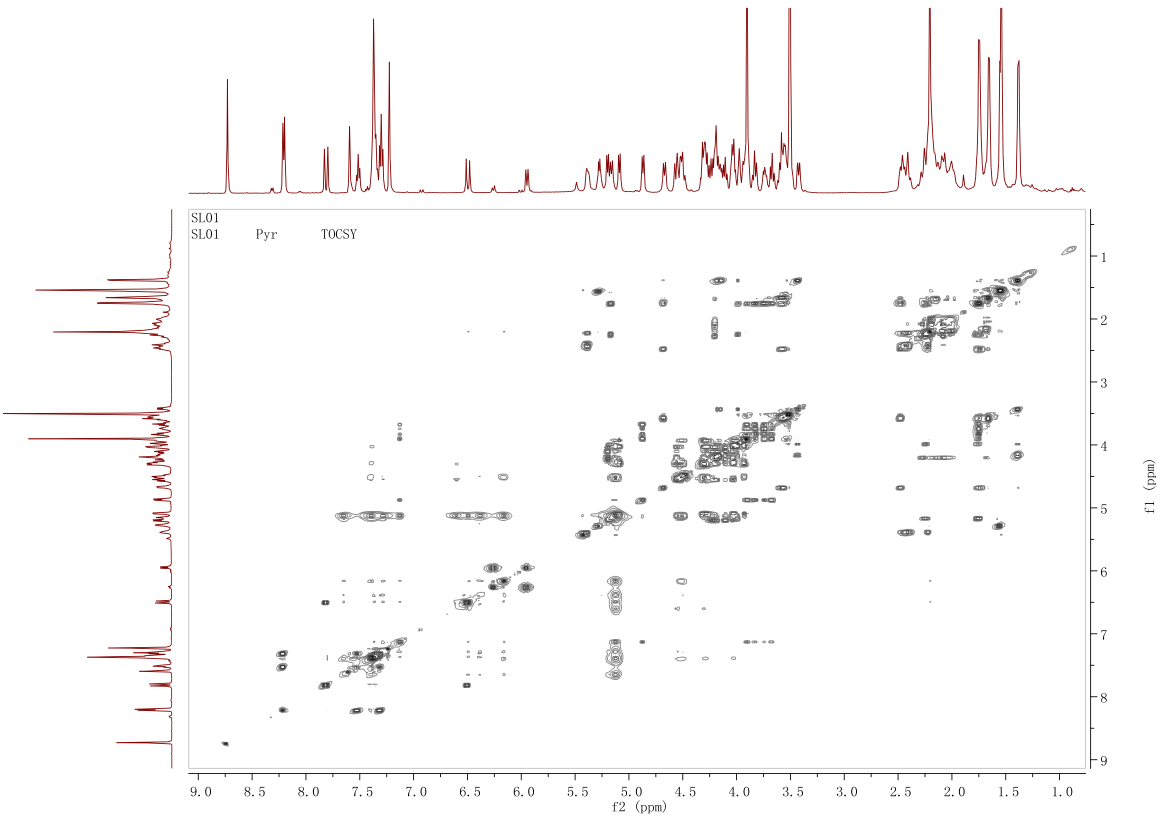


**Figure S7.** TOCSY (500 MHz) spectrum of **1** in Pyridine-$d_5$.

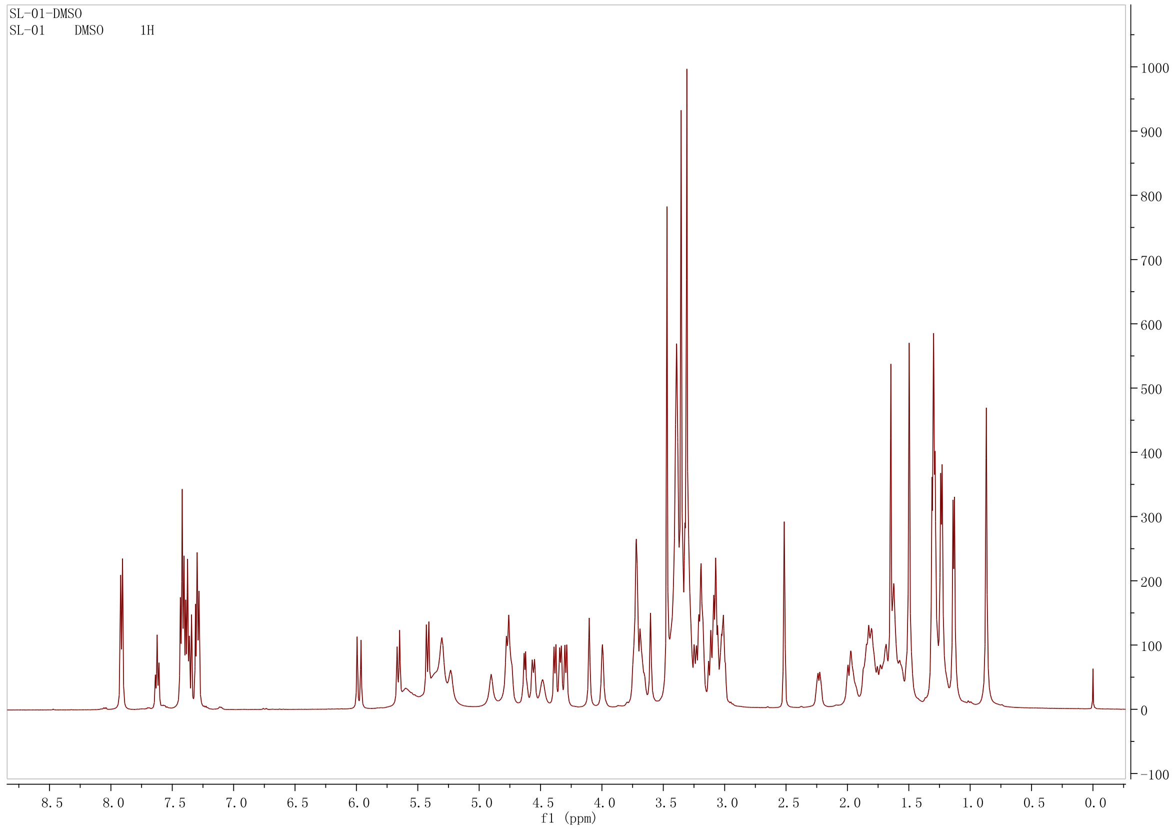


**Figure S8.** $^1$H-NMR (500 MHz) spectrum of **1** in DMSO-$d_6$.

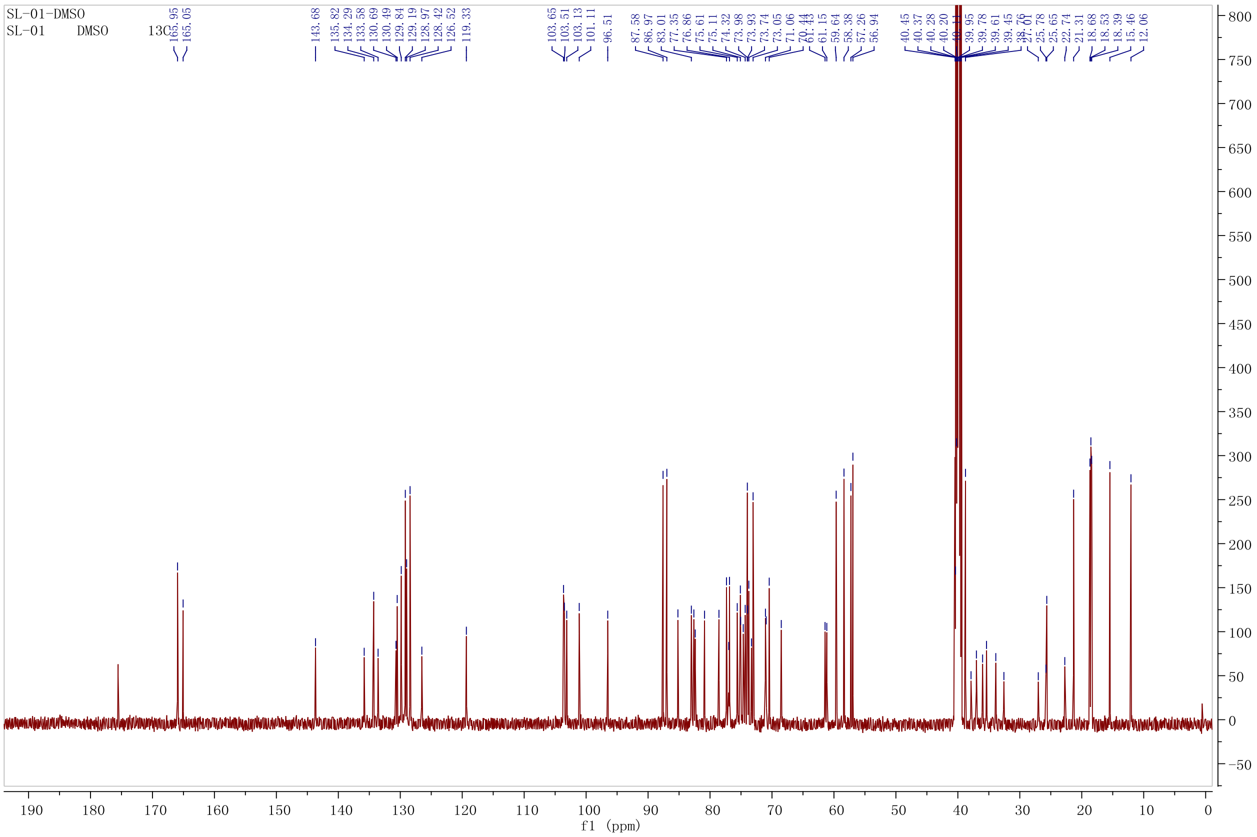


**Figure S9.** $^{13}$C-NMR (125 MHz) spectrum of **1** in DMSO-*d*6.

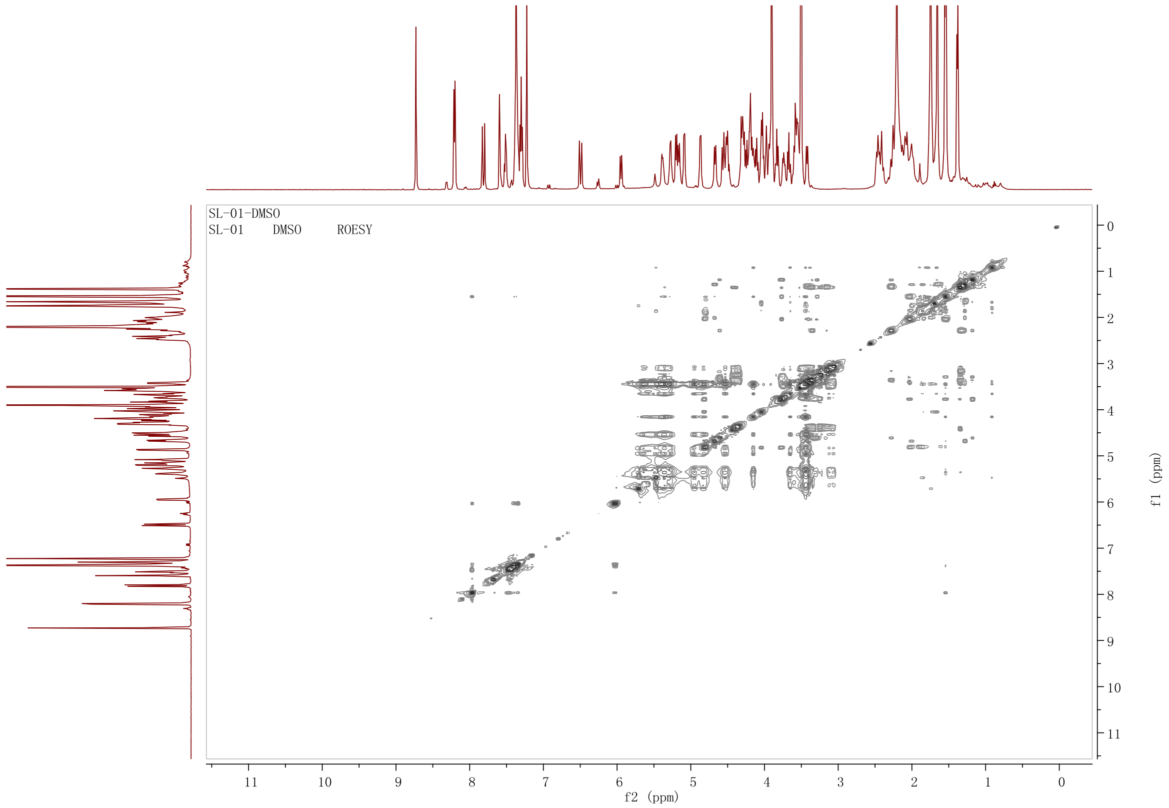


**Figure S10.** ROESY (500 MHz) spectrum of **1** in DMSO-*d*6.

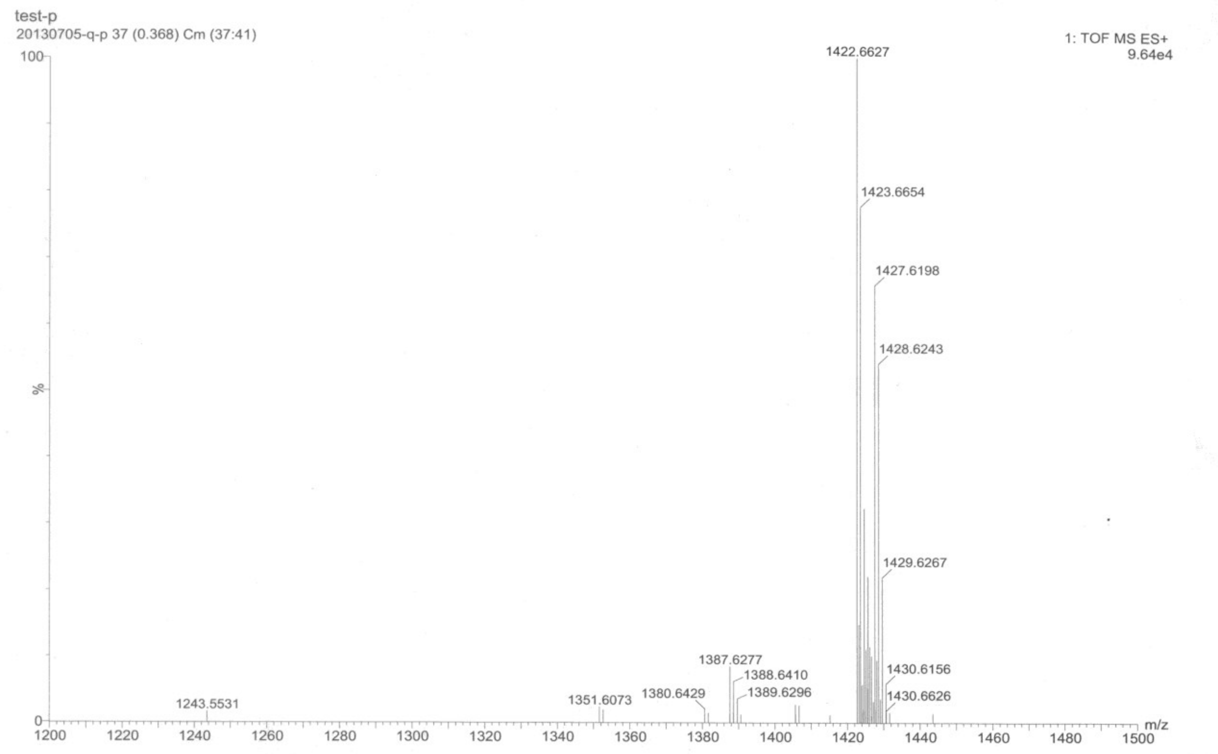


**Figure S11.** HRESIMS spectrum of **1**.

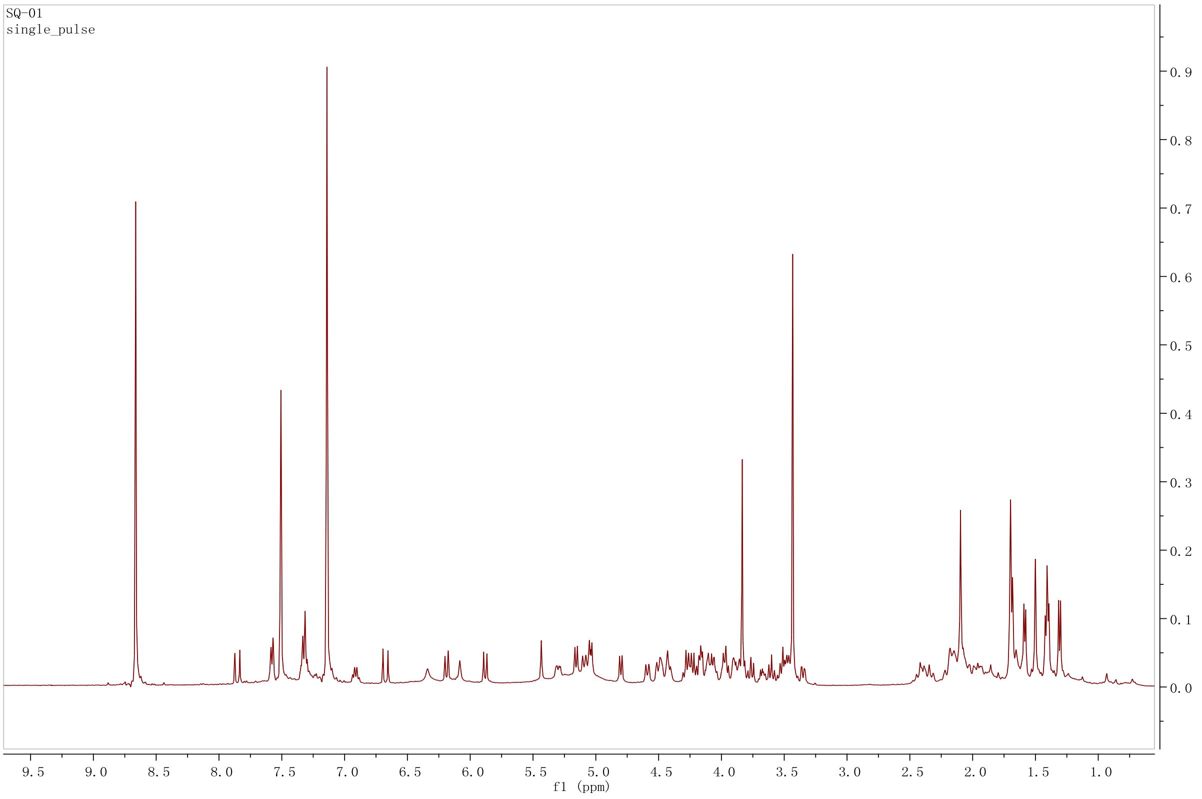


**Figure S12.** $^{1}$H-NMR (400 MHz) spectrum of **2** in Pyridine-$d_5$.

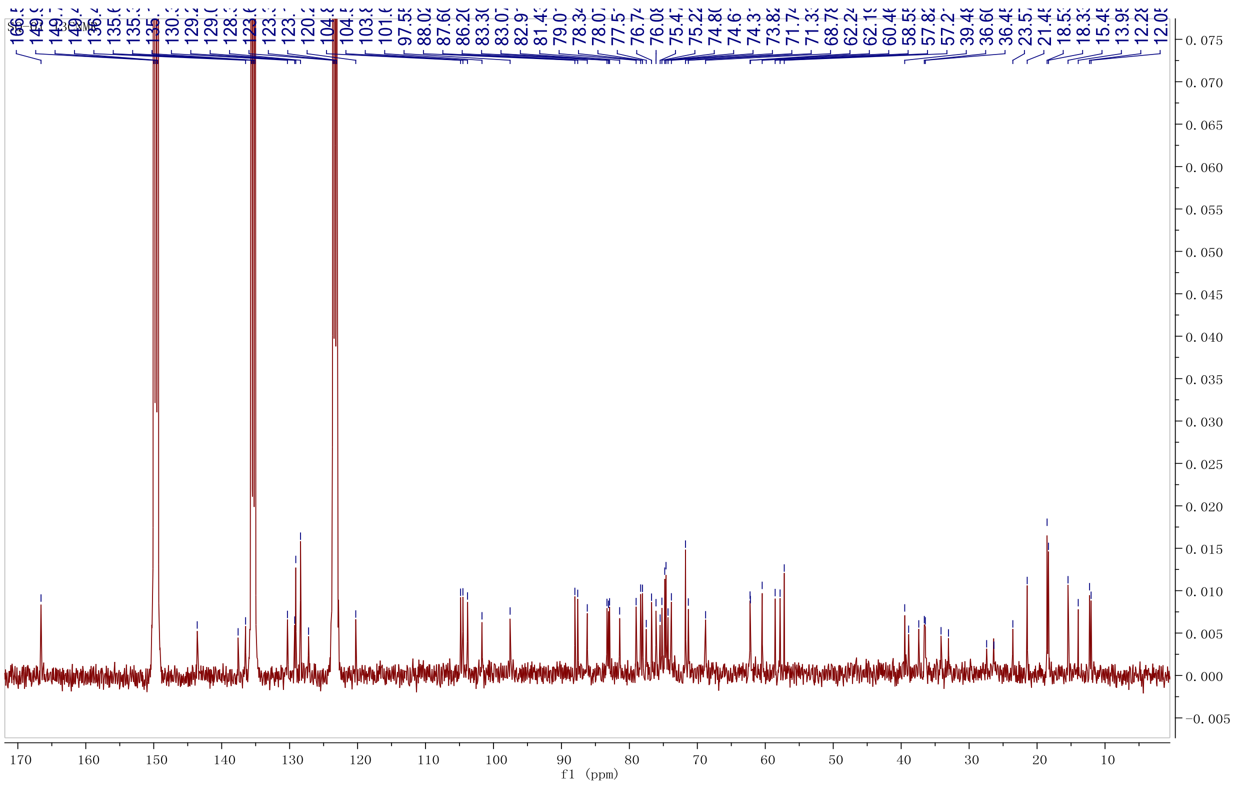

**Figure S13.** ${}^{13}$C-NMR (100 MHz) spectrum of **2** in Pyridine-$d_5$.

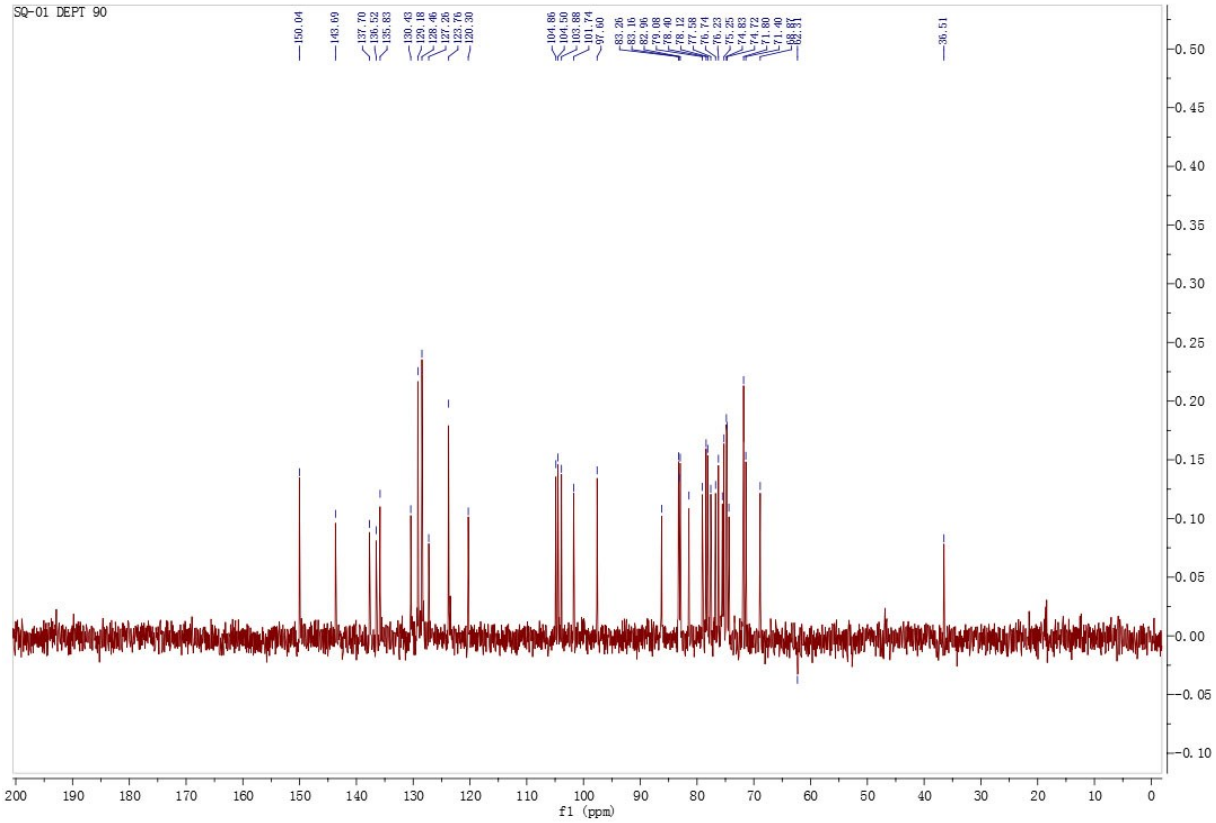


**Figure S14.** DEPT (θ = 90°) (400 MHz) spectrum of **2** in Pyridine-$d_5$.

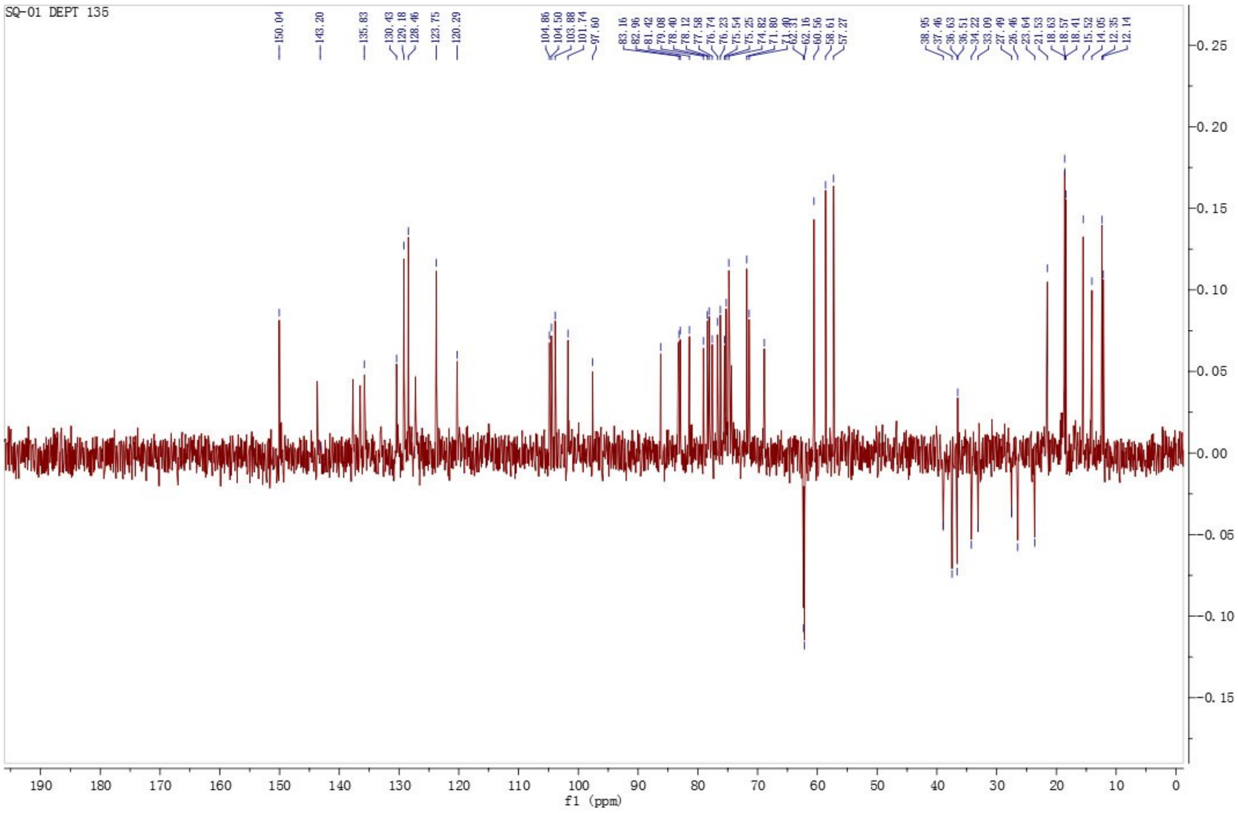


**Figure S15.** DEPT (θ = 135°) (400 MHz) spectrum of **2** in Pyridine-*d*5.

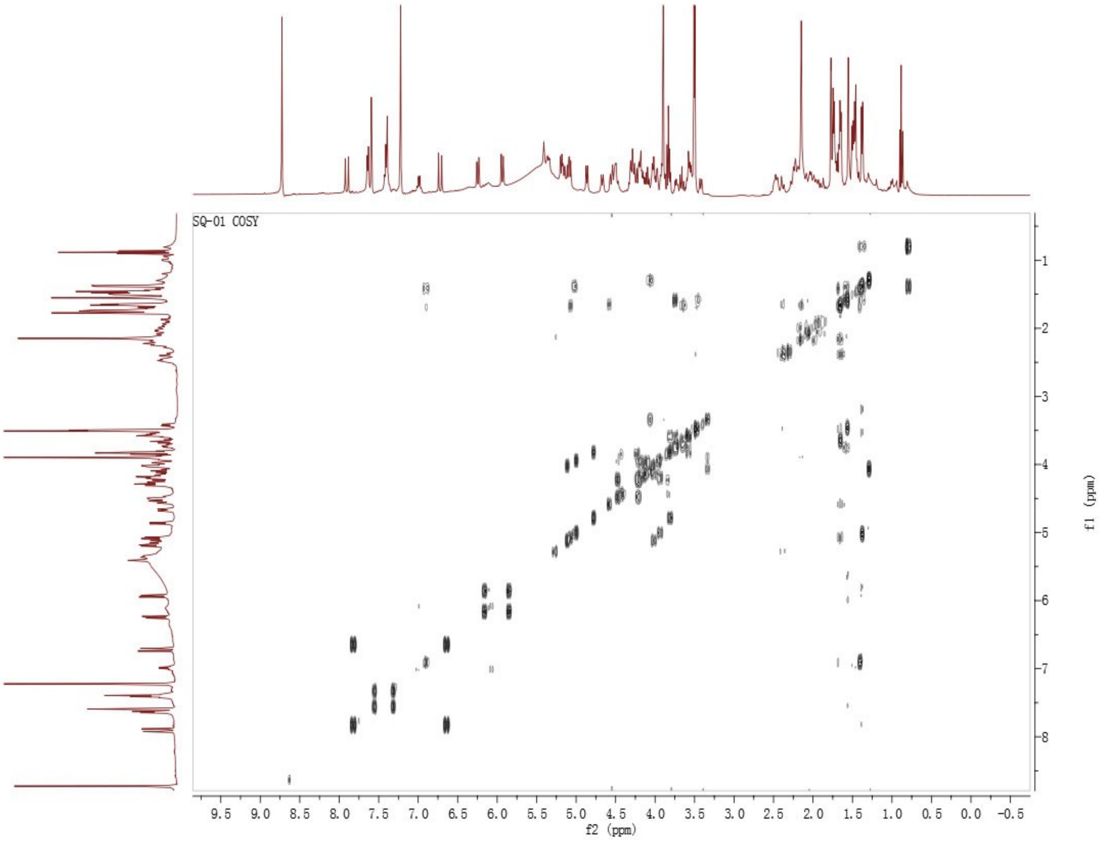


**Figure S16.** COSY (400 MHz) spectrum of **2** in Pyridine-*d*5.

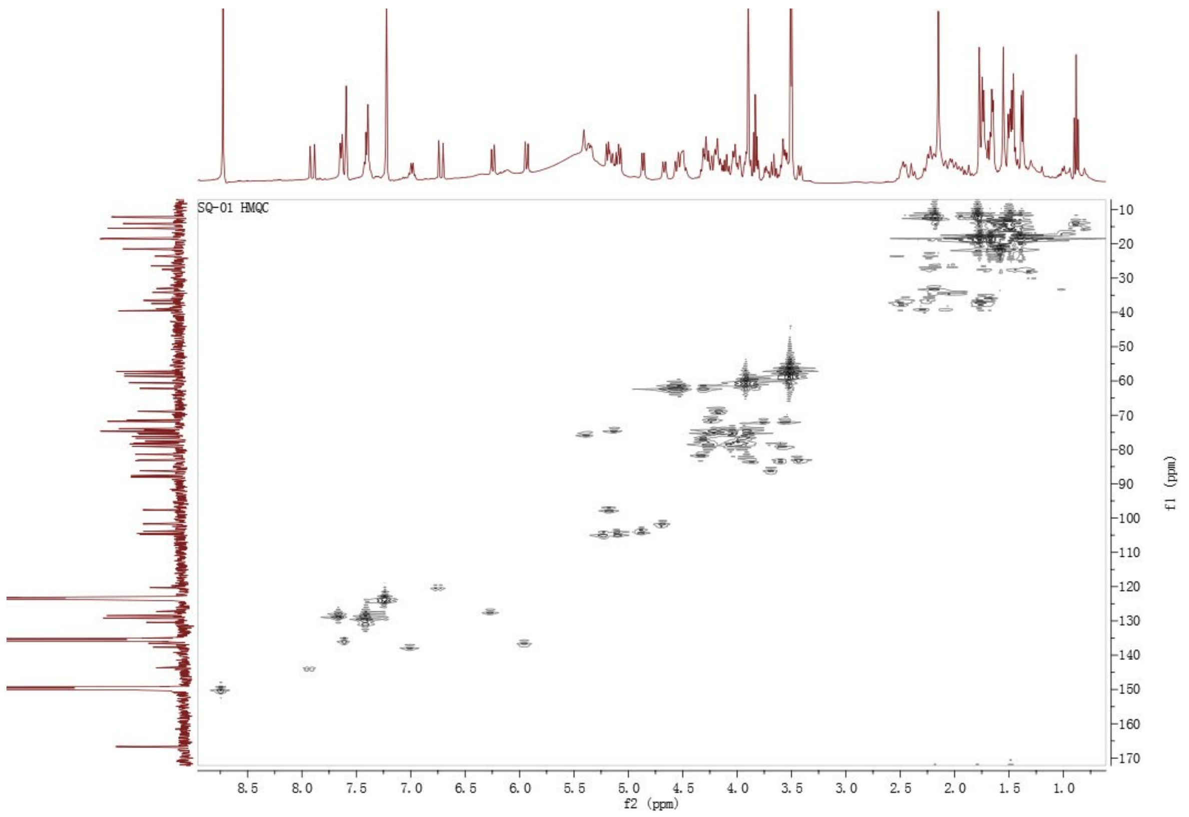


**Figure S17.** HMQC spectrum of **2** in Pyridine-$d_5$.

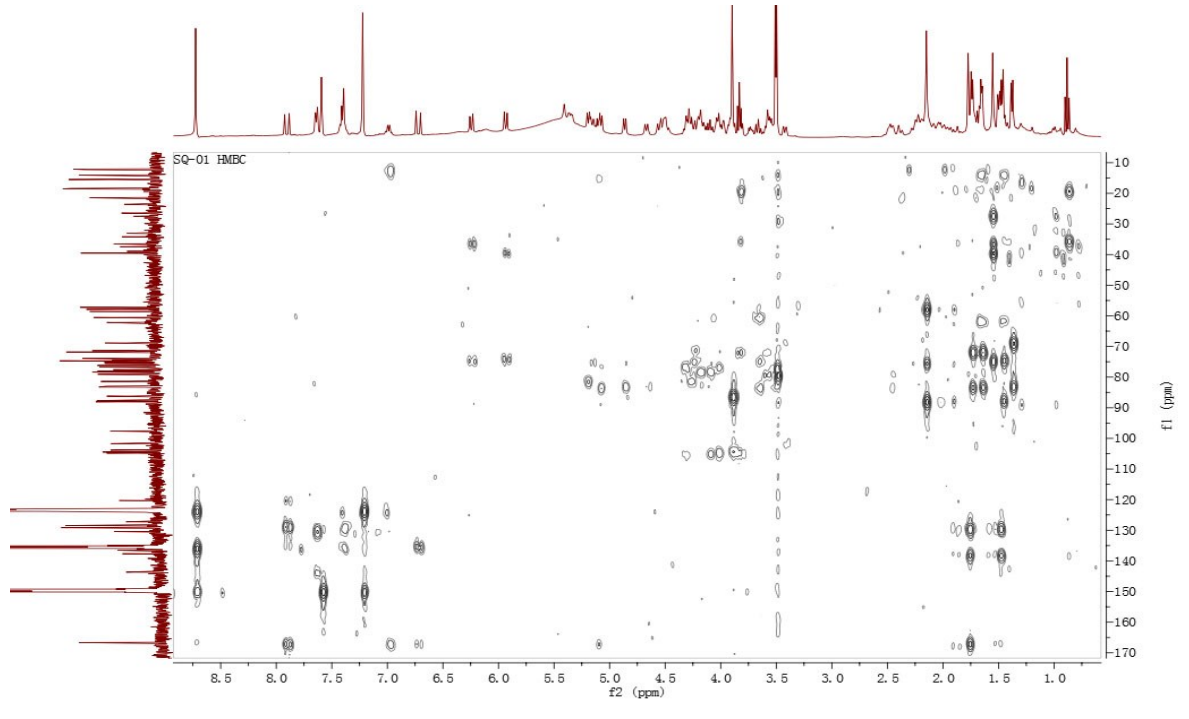


**Figure S18.** HMBC spectrum of **2** in Pyridine-$d_5$.

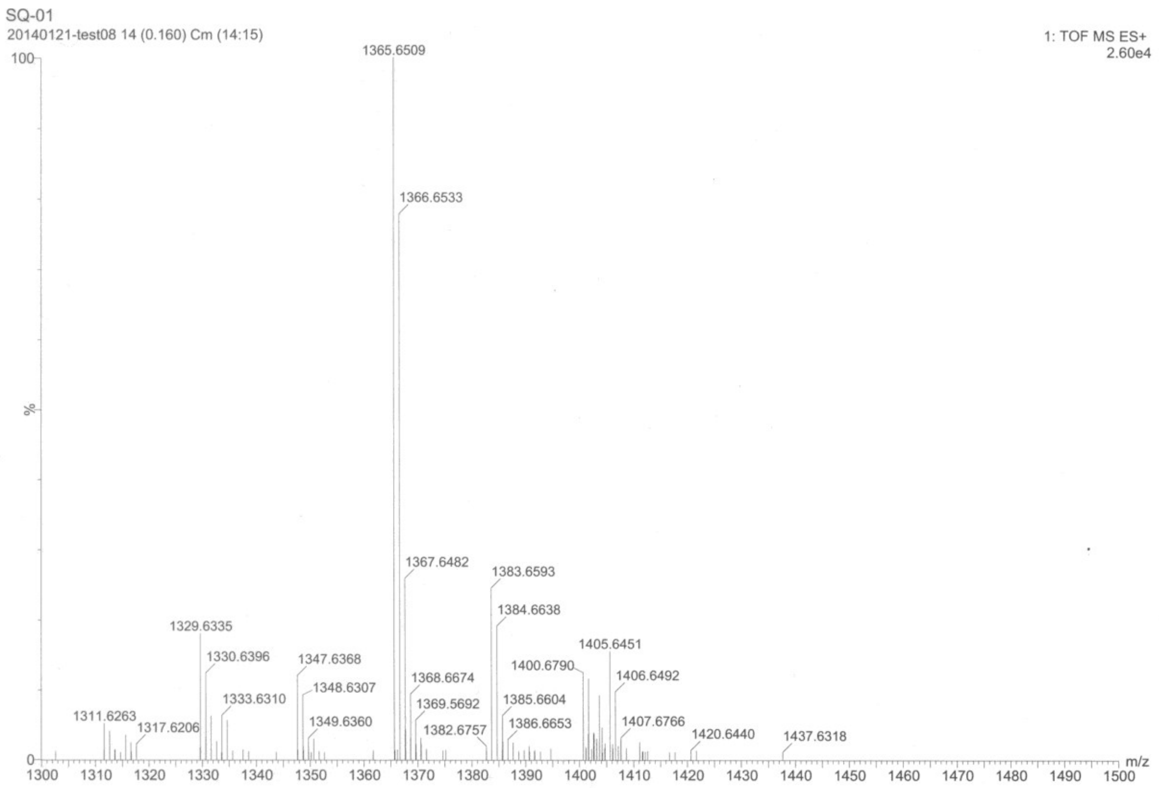


**Figure S19.** HRESIMS spectrum of 2.

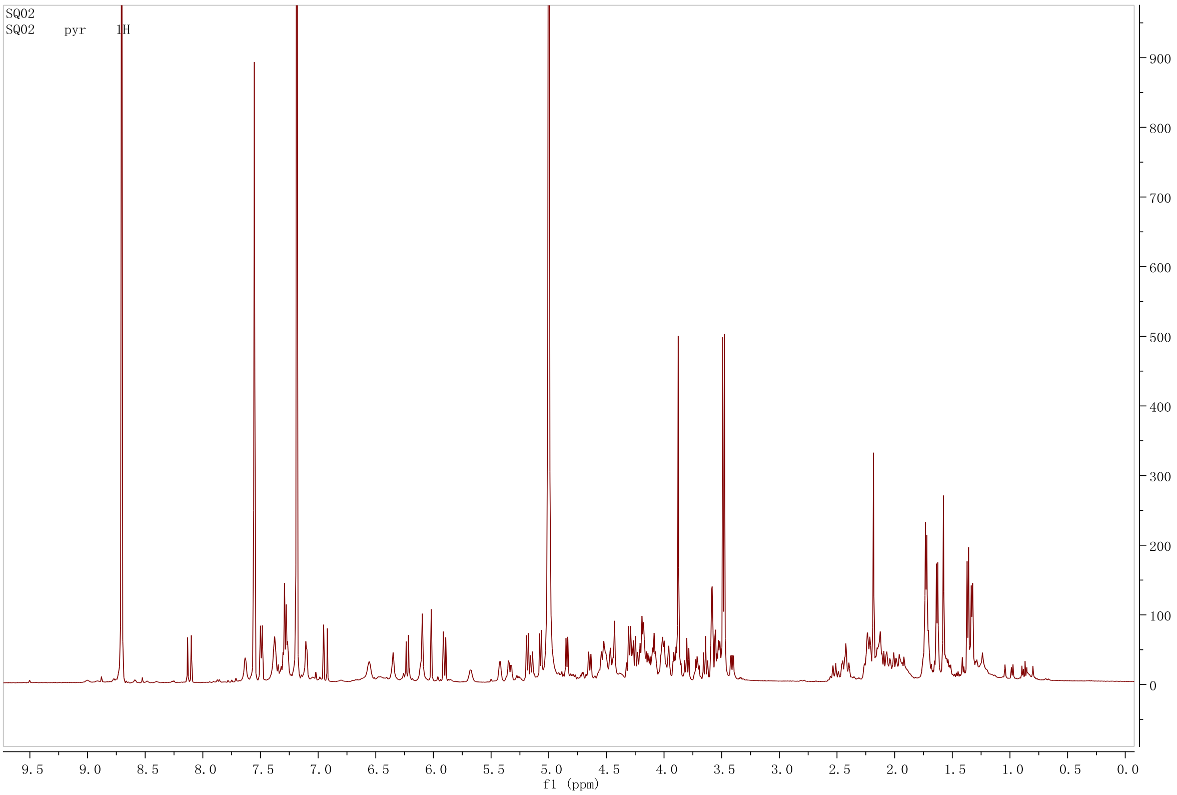


**Figure S20.** $^{1}$H-NMR (500 MHz) spectrum of **3** in Pyridine-$d_5$.

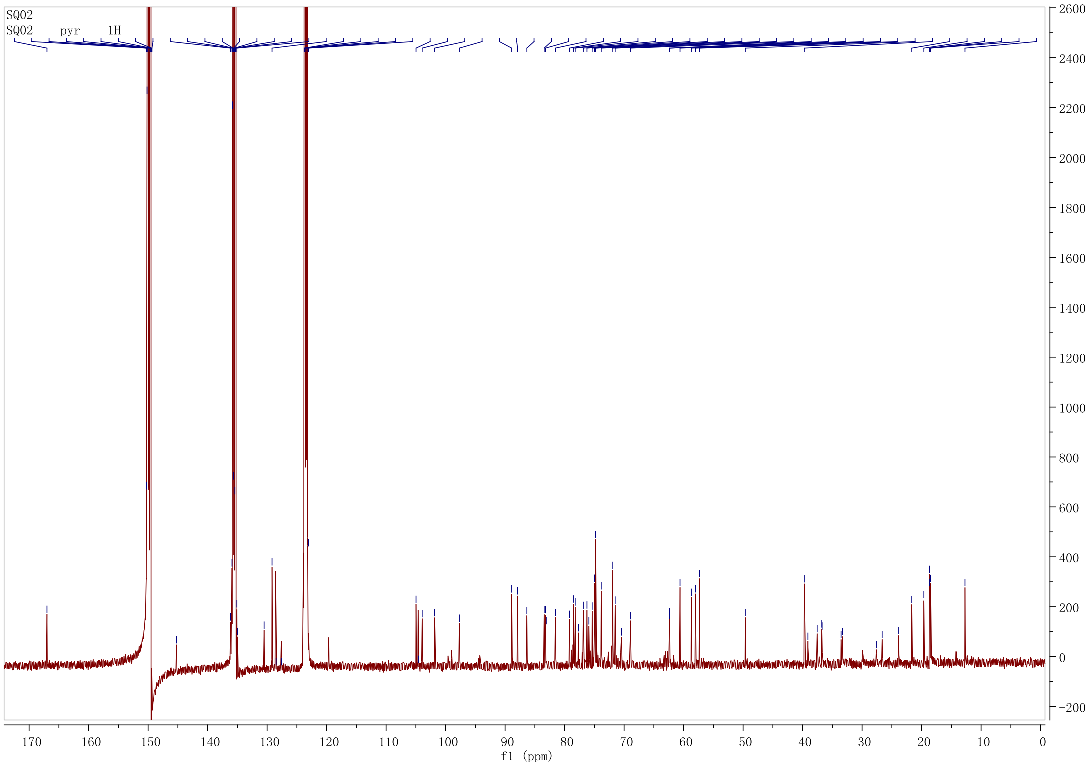

**Figure S21.** $^{13}$C-NMR (125 MHz) spectrum of **3** in Pyridine-$d_5$.

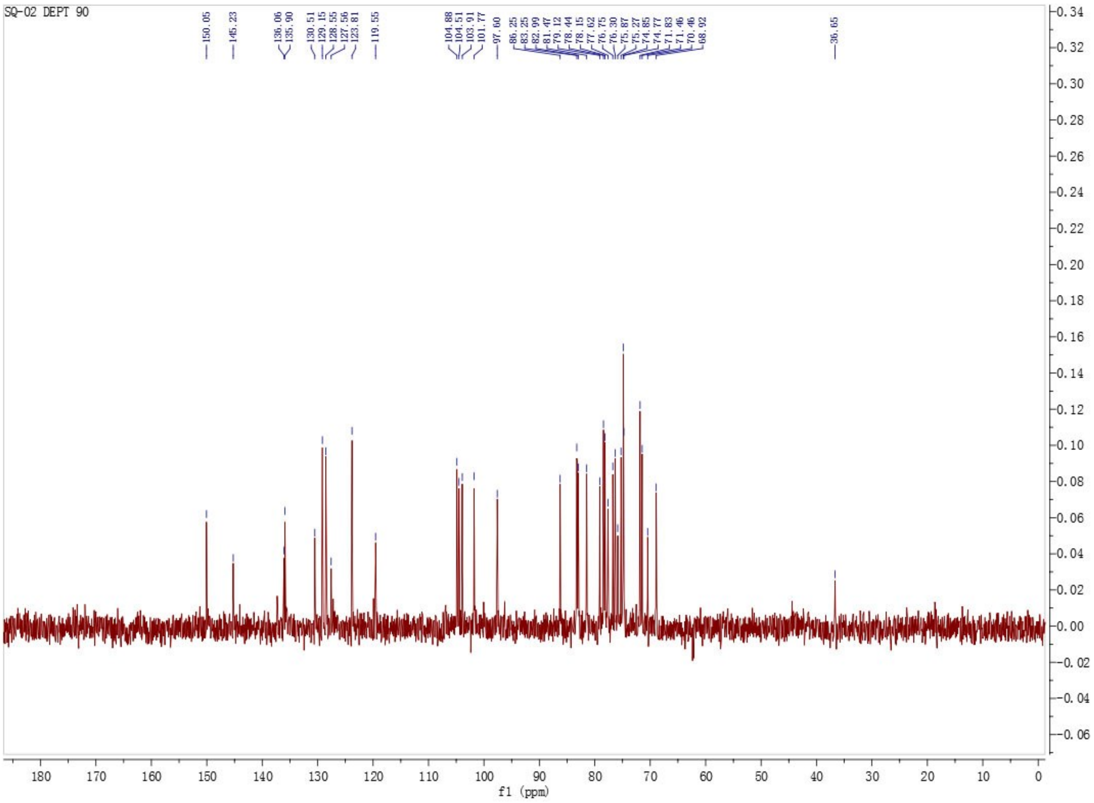

**Figure S22.** DEPT (θ = 90°) (400 MHz) spectrum of **3** in Pyridine-$d_5$.

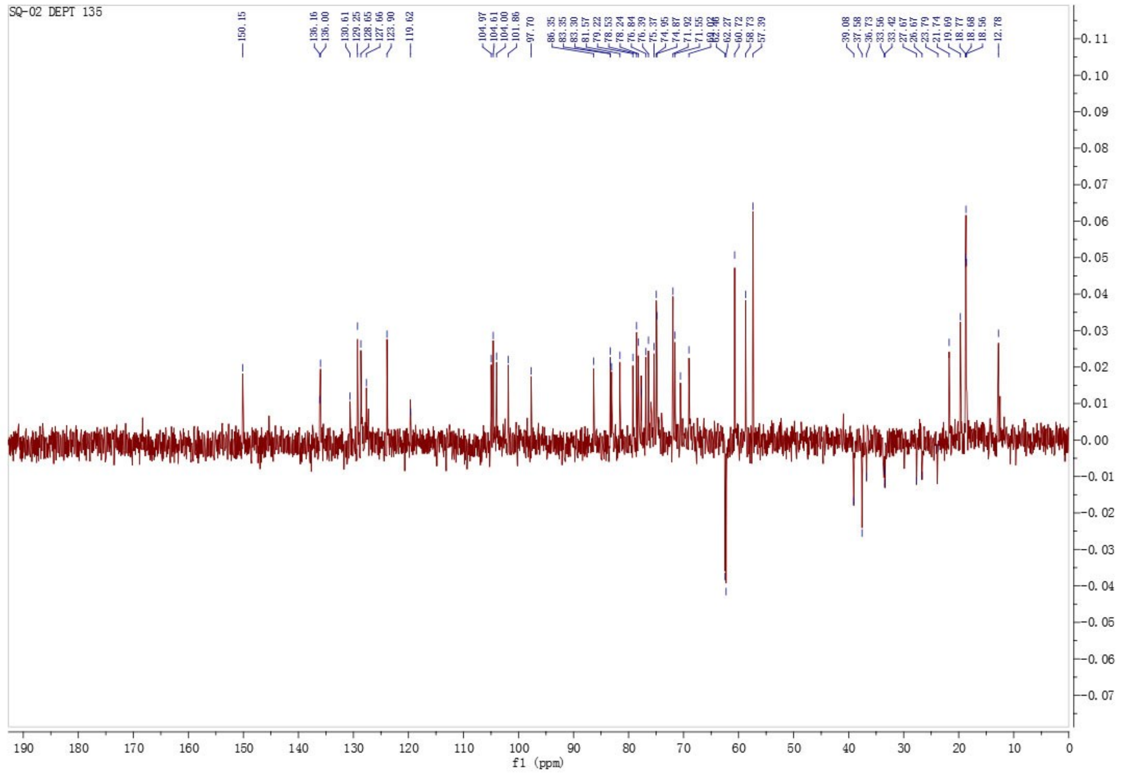

**Figure S23.** DEPT (θ = 135°) (400 MHz) spectrum of **3** in Pyridine-$d_5$.

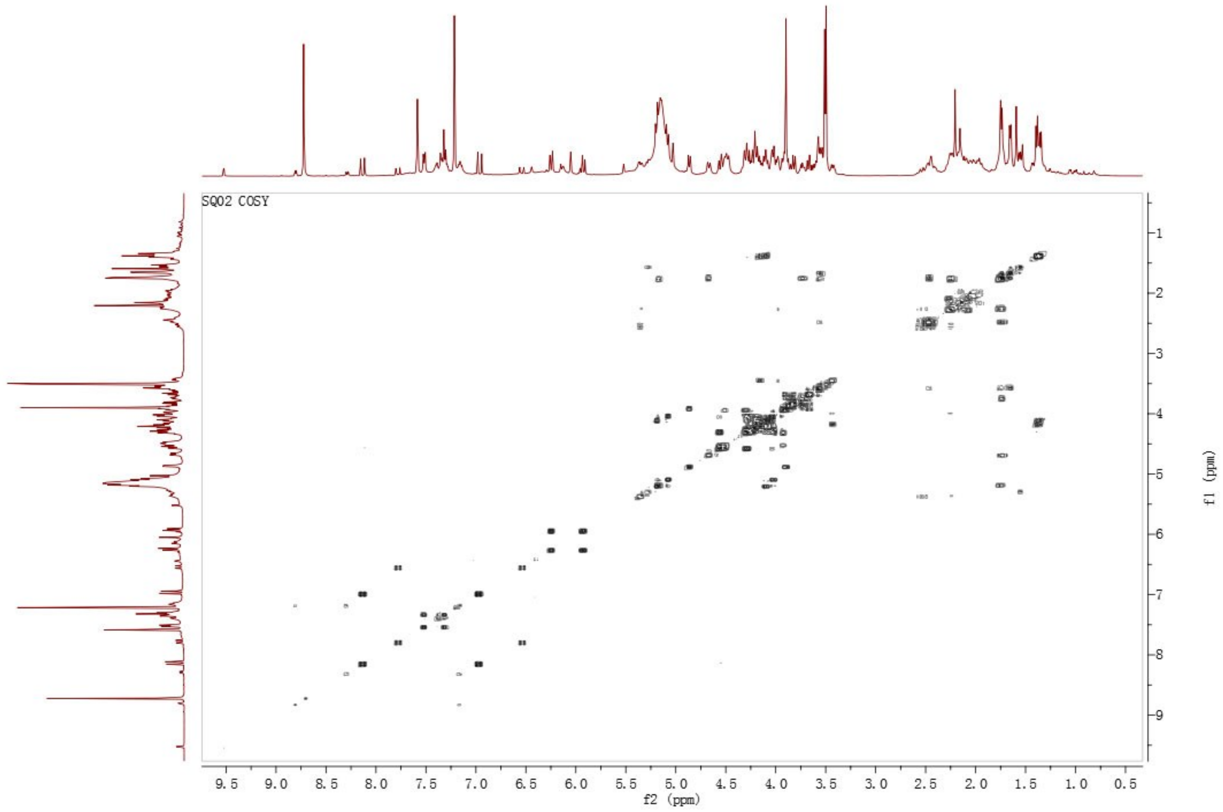

**Figure S24.** COSY (400 MHz) spectrum of **3** in Pyridine-$d_5$.

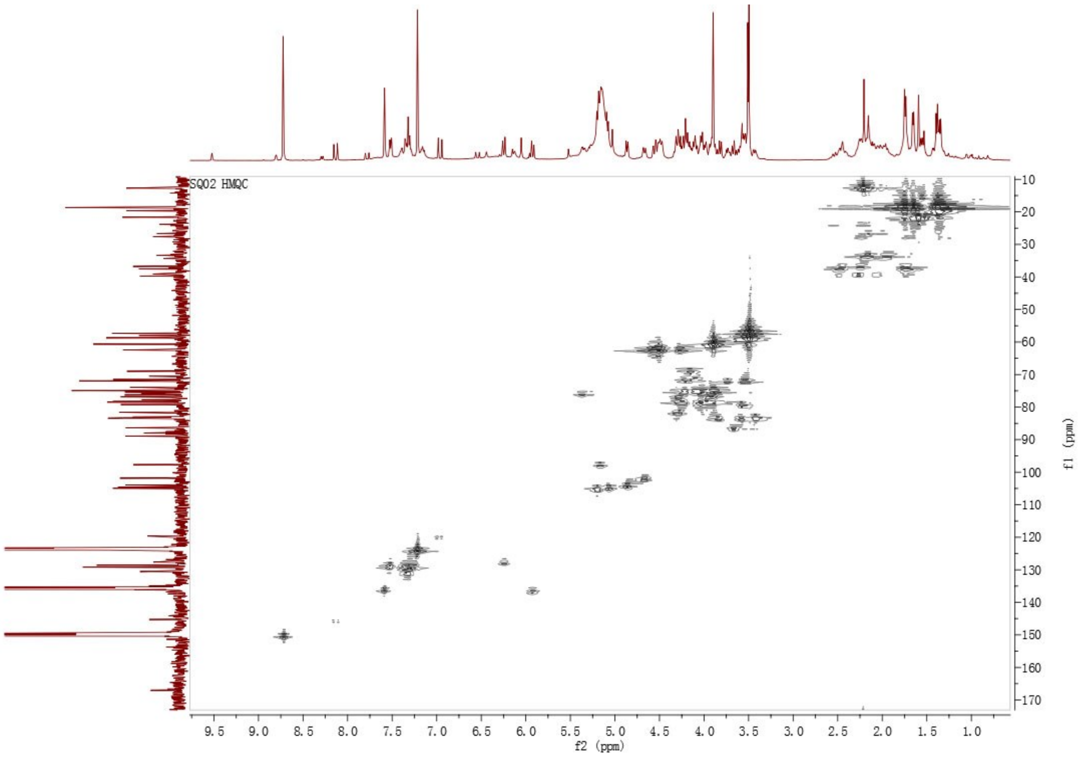


**Figure S25.** HMQC spectrum of **3** in Pyridine-$d_5$.

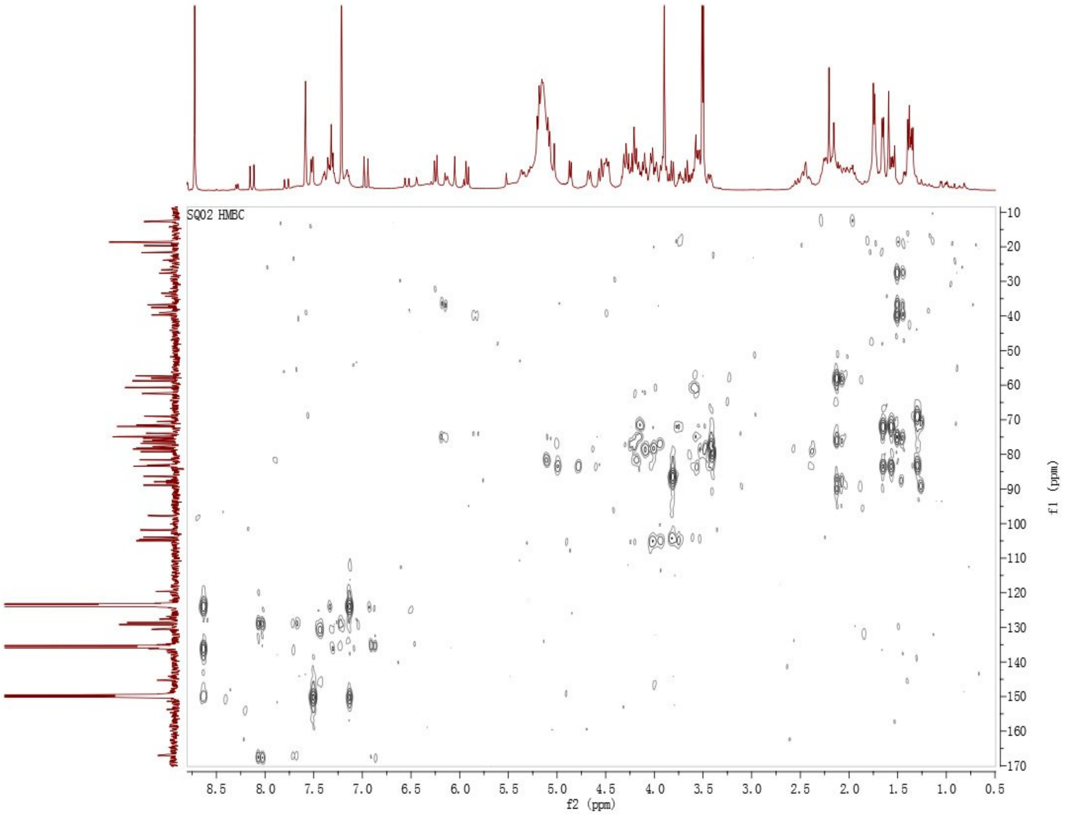


**Figure S26.** HMBC spectrum of **3** in Pyridine-$d_5$.

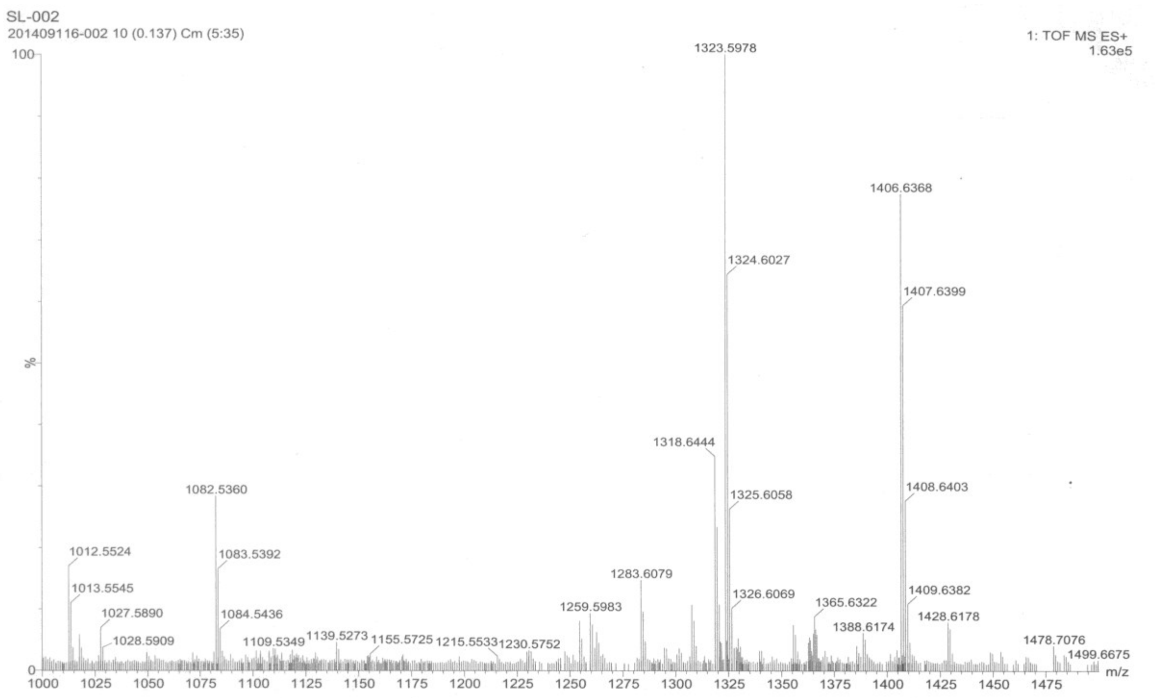


**Figure S27.** HRESIMS spectrum of **3**.

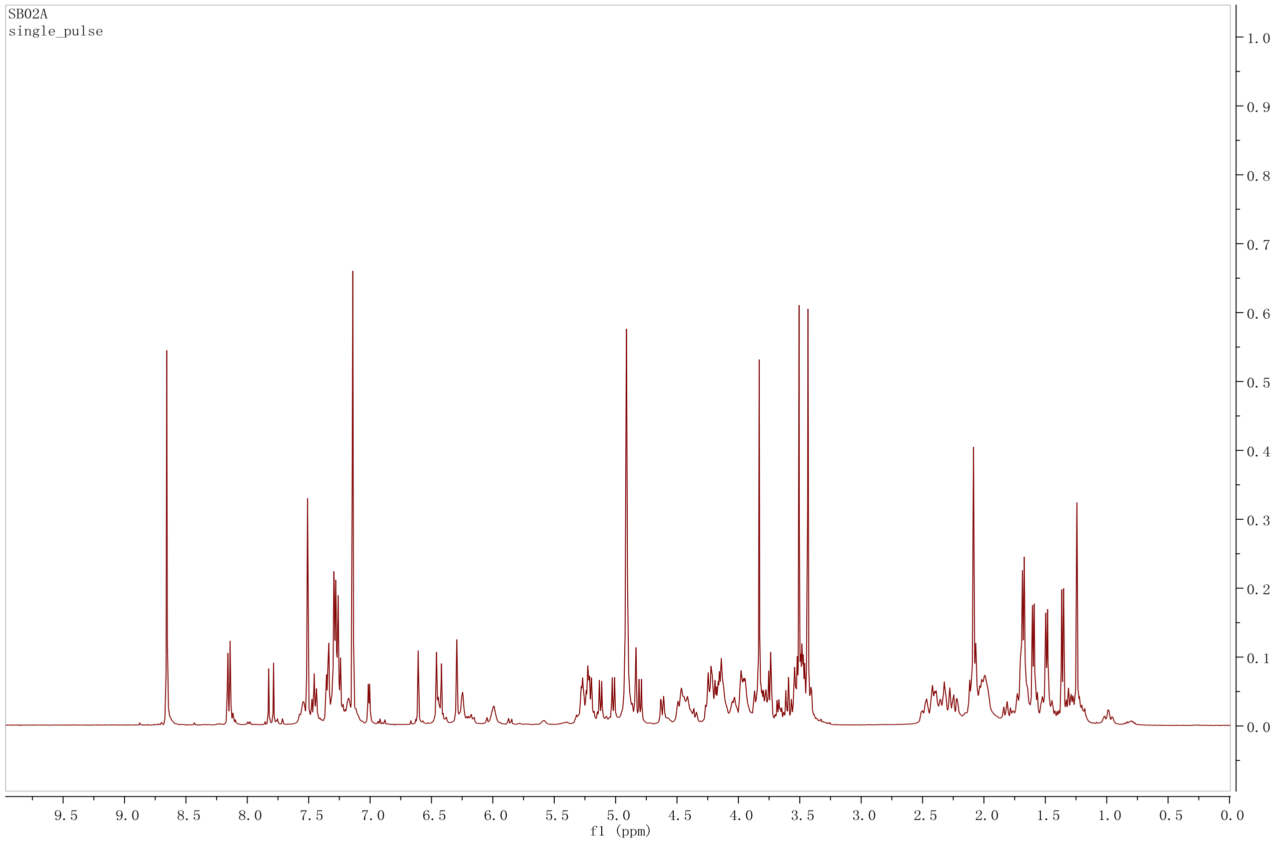


**Figure S28.** $^{1}$H-NMR (400 MHz) spectrum of **4** in Pyridine-$d_5$.

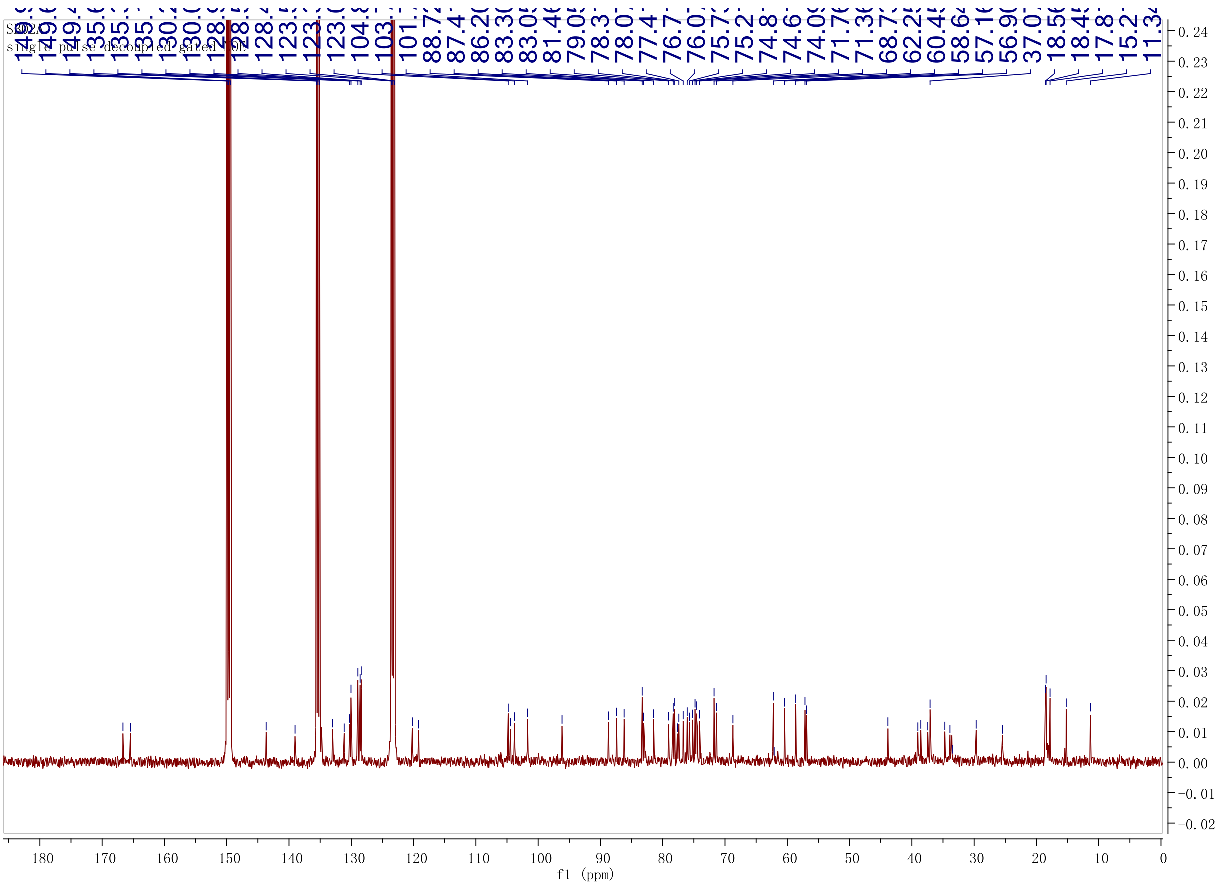


**Figure S29.** $^{13}$C-NMR (100 MHz) spectrum of **4** in Pyridine-$d_5$.

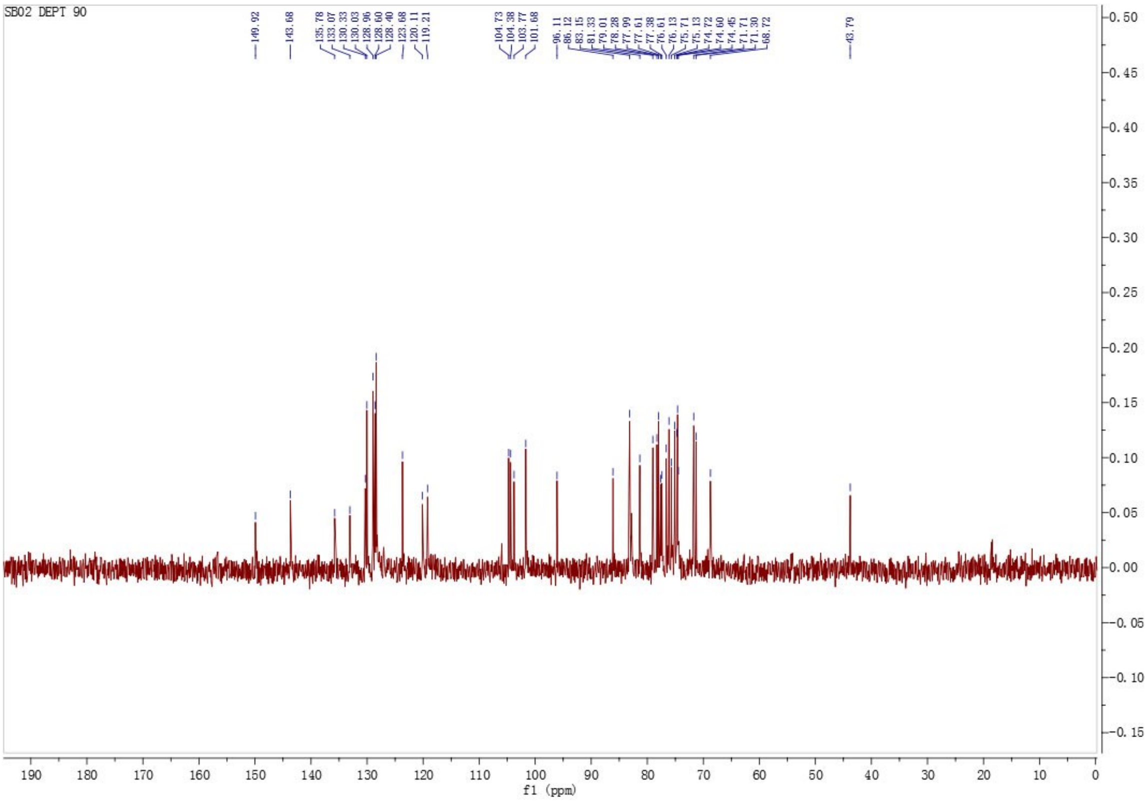


**Figure S30.** DEPT (θ = 90°) (400 MHz) spectrum of **4** in Pyridine-$d_5$.

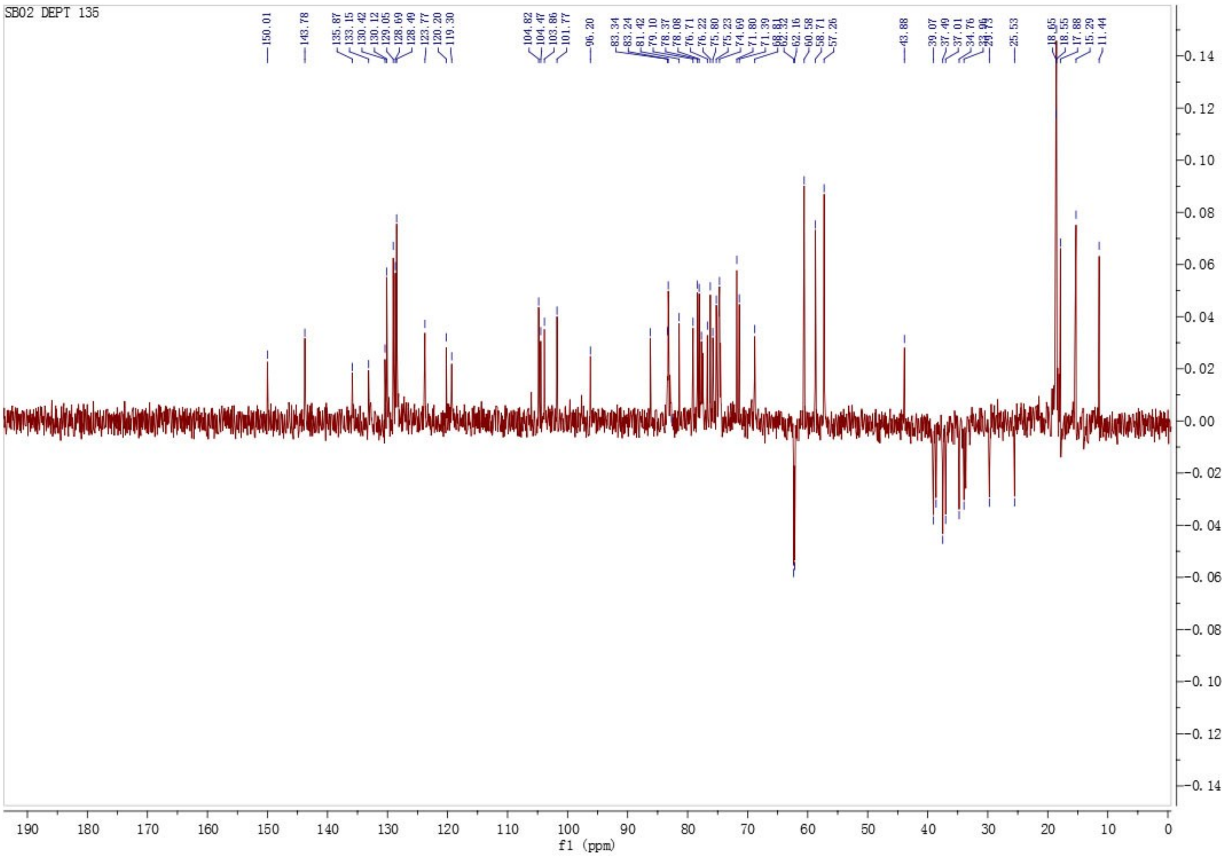


**Figure S31.** DEPT (θ = 135°) (400 MHz) spectrum of **4** in Pyridine-$d_5$.

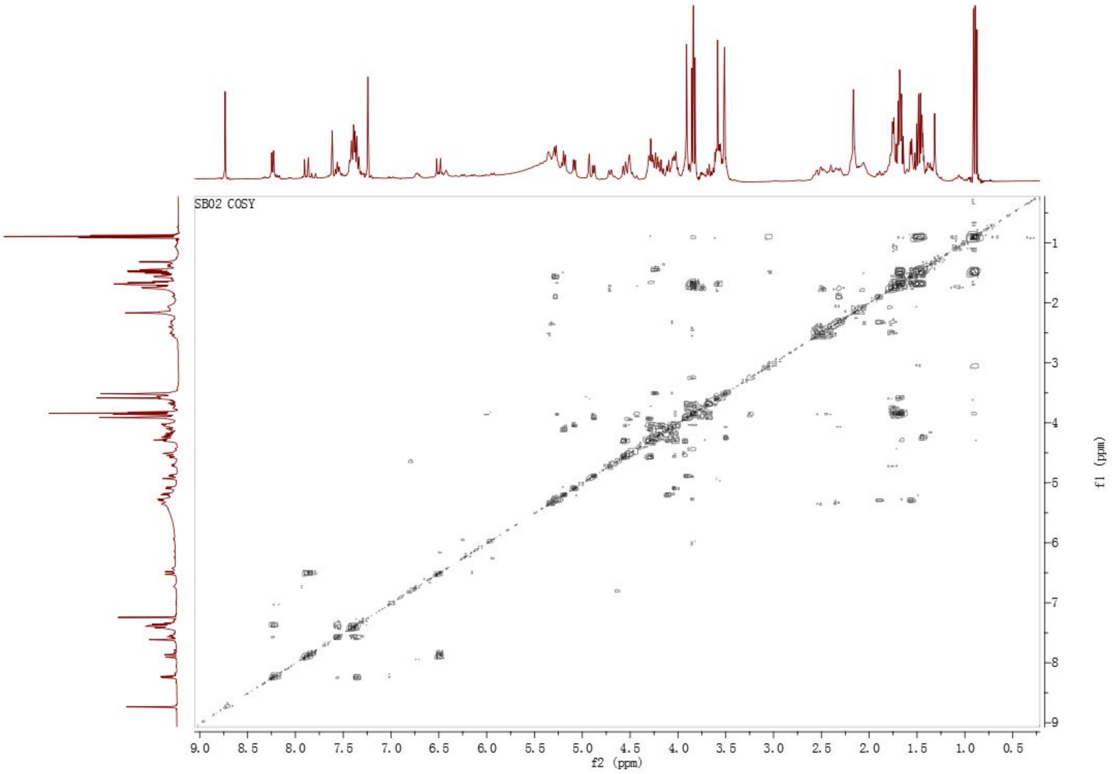


**Figure S32.** COSY (400 MHz) spectrum of **4** in Pyridine-$d_5$.

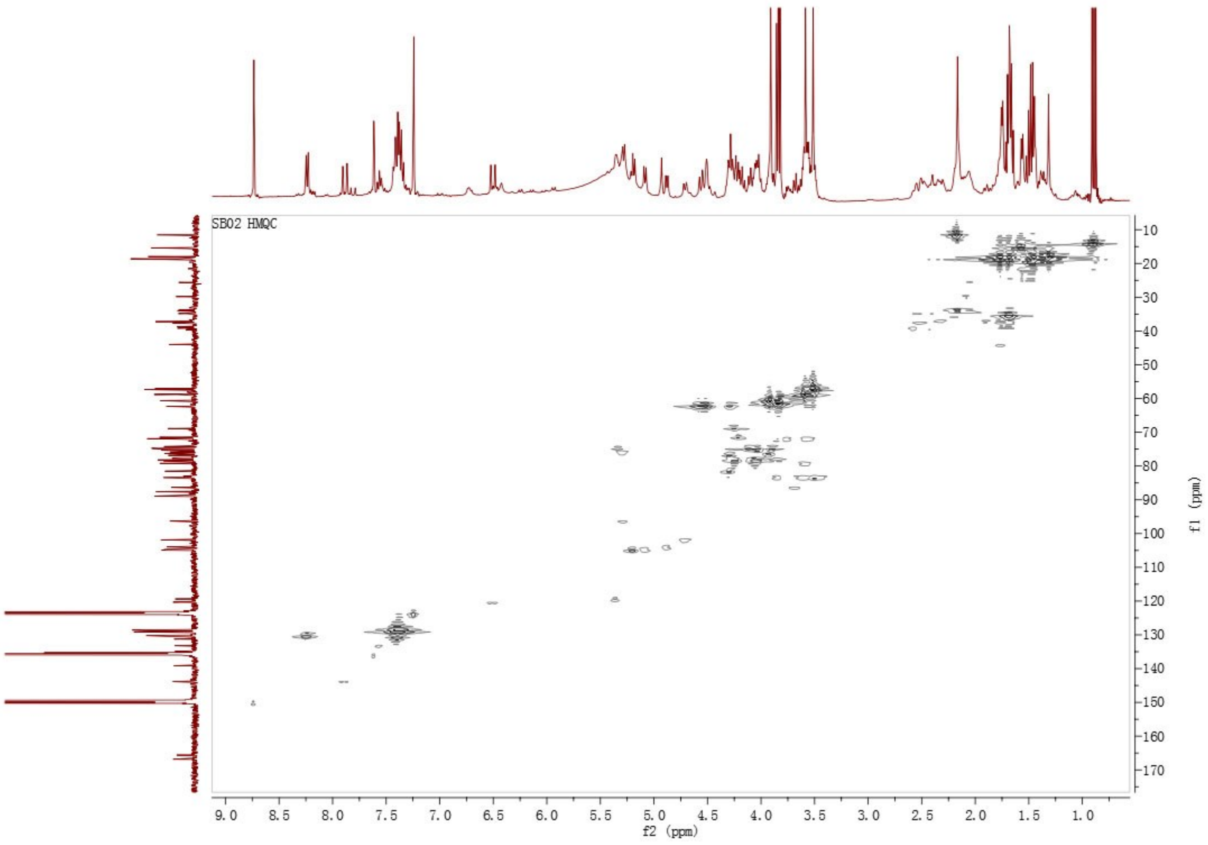


**Figure S33.** HMQC spectrum of **4** in Pyridine-$d_5$.

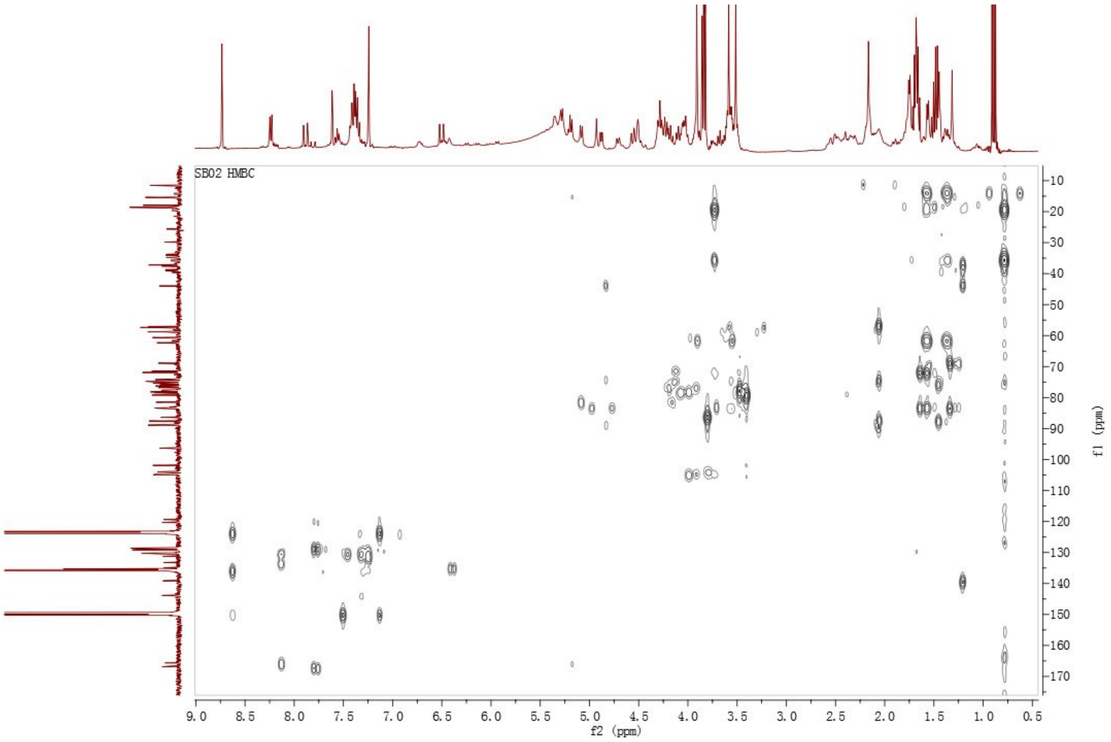


**Figure S34.** HMBC spectrum of **4** in Pyridine-$d_5$.

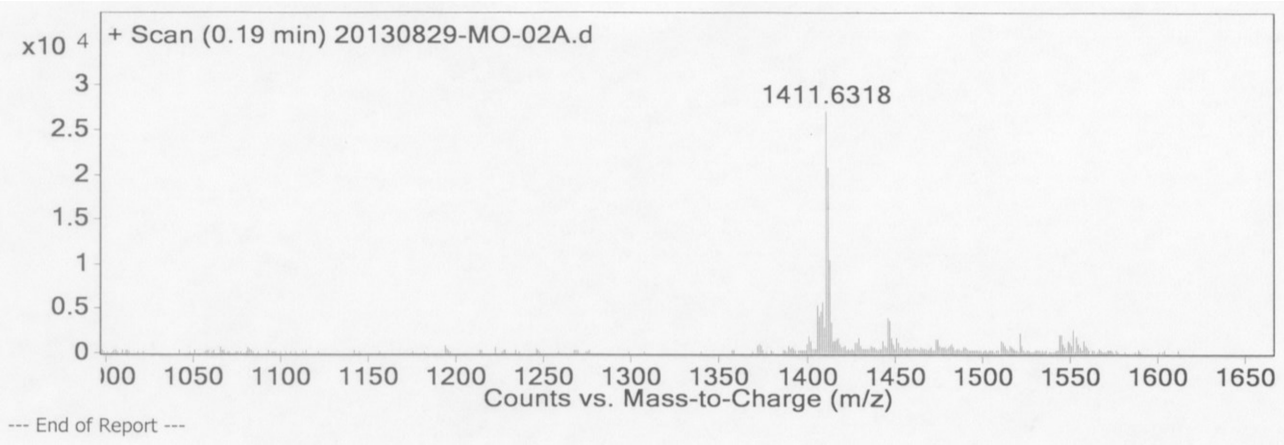


**Figure S35.** HRESIMS spectrum of **4**.

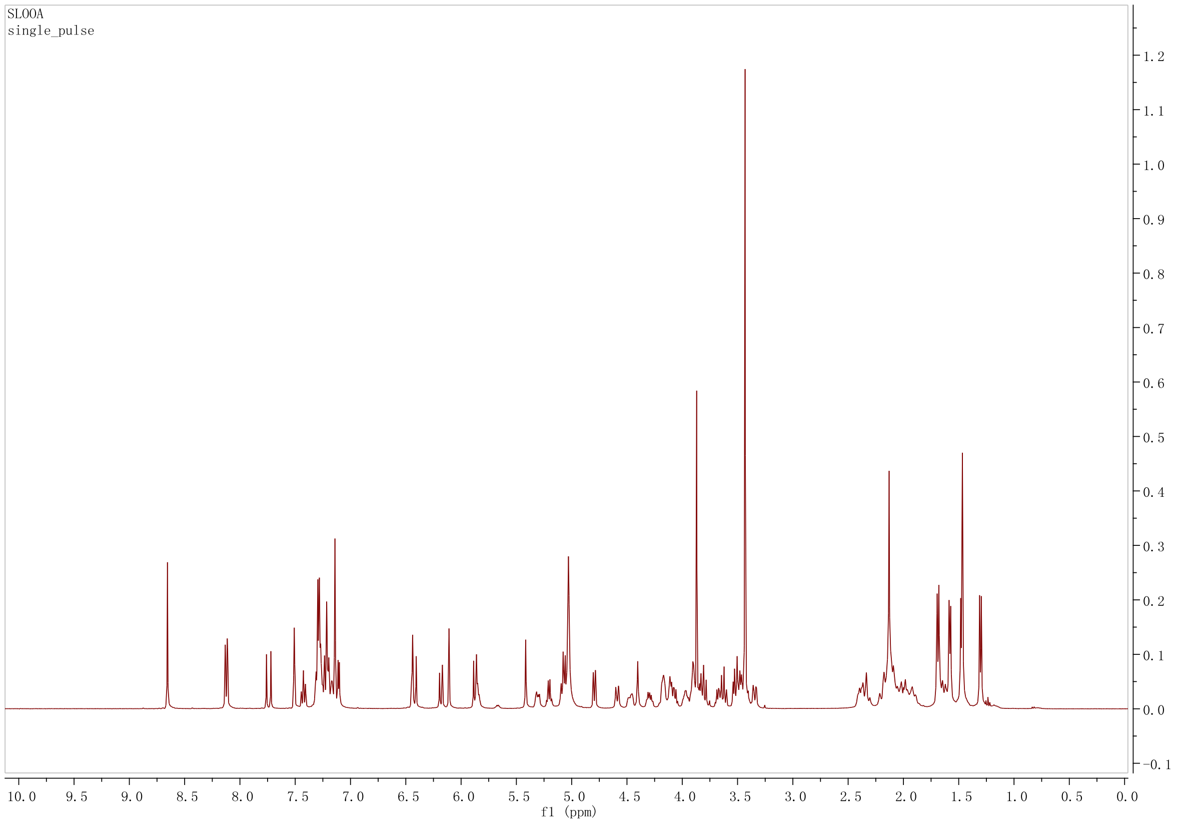


**Figure S36.** $^{1}$H-NMR (400 MHz) spectrum of **5** in Pyridine-$d_5$.

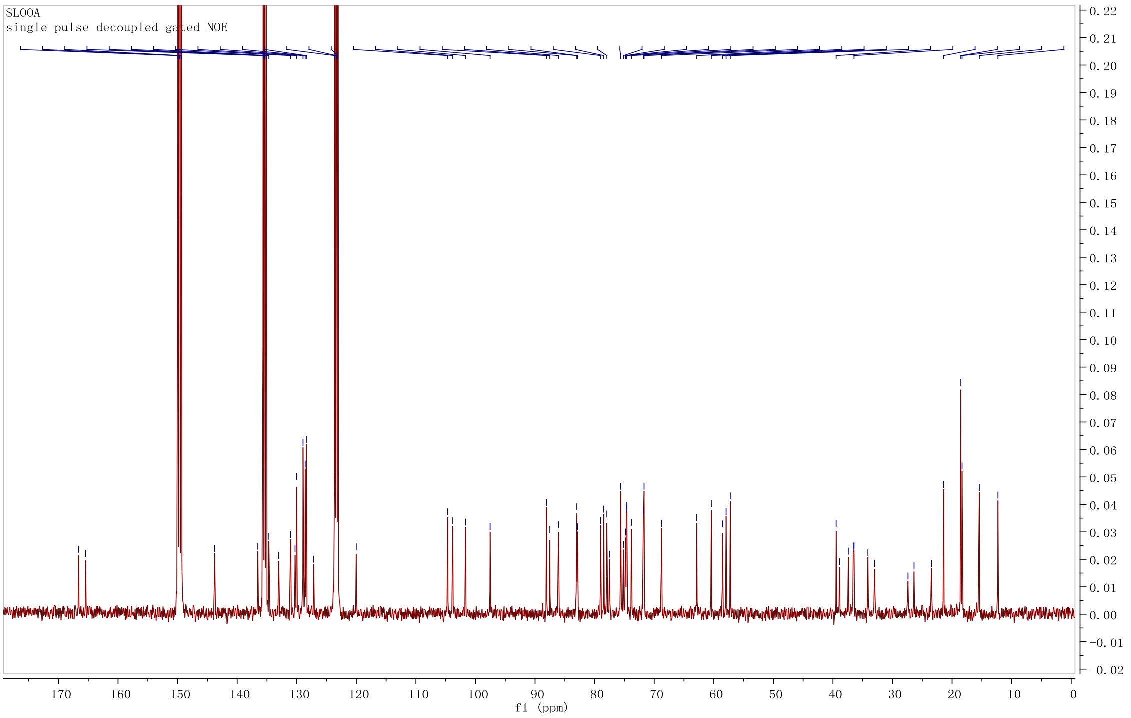


**Figure S37.** $^{13}$C-NMR (100 MHz) spectrum of **5** in Pyridine-$d_5$.

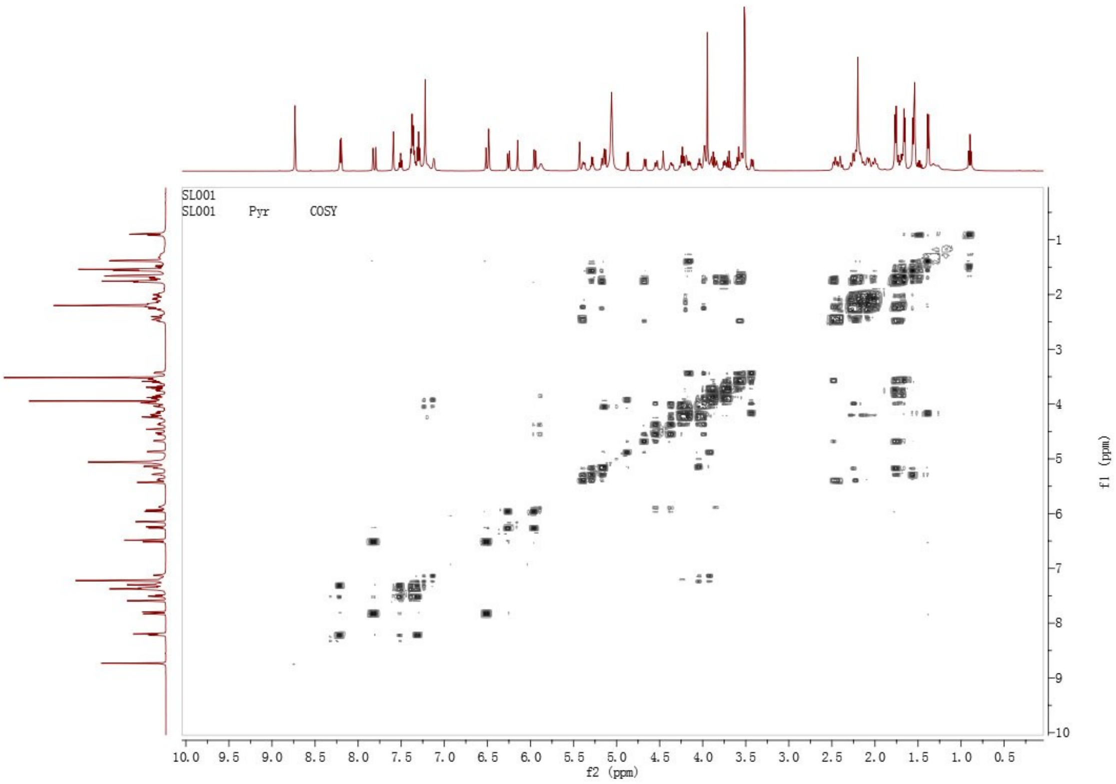


**Figure S38.** COSY (500 MHz) spectrum of **5** in Pyridine-$d_5$.

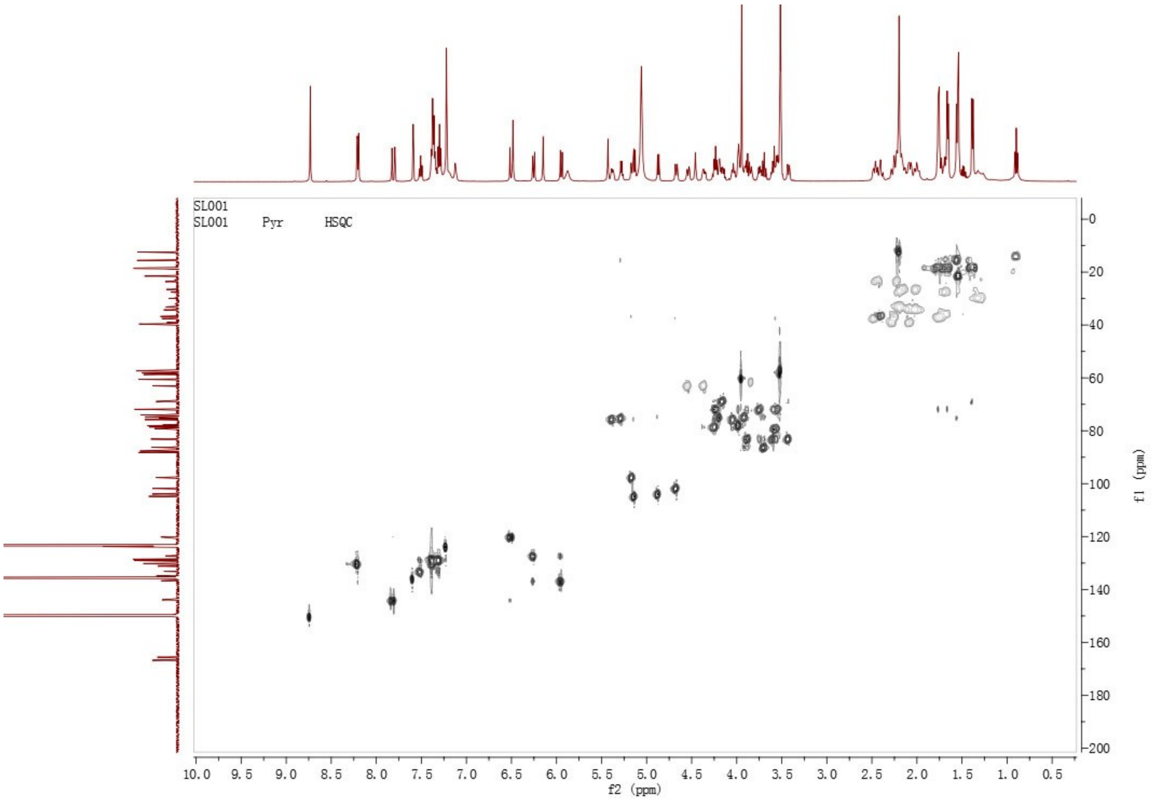


**Figure S39.** HMQC spectrum of **5** in Pyridine-$d_5$.

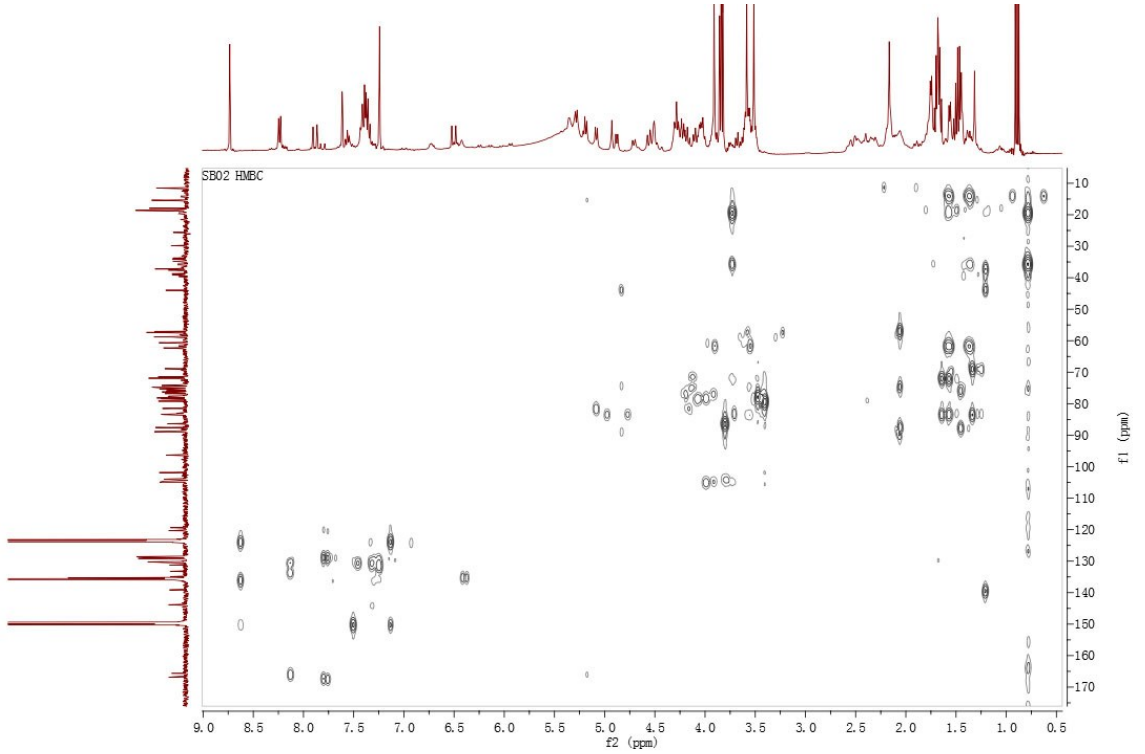


**Figure S40.** HMBC spectrum of **5** in Pyridine-$d_5$.

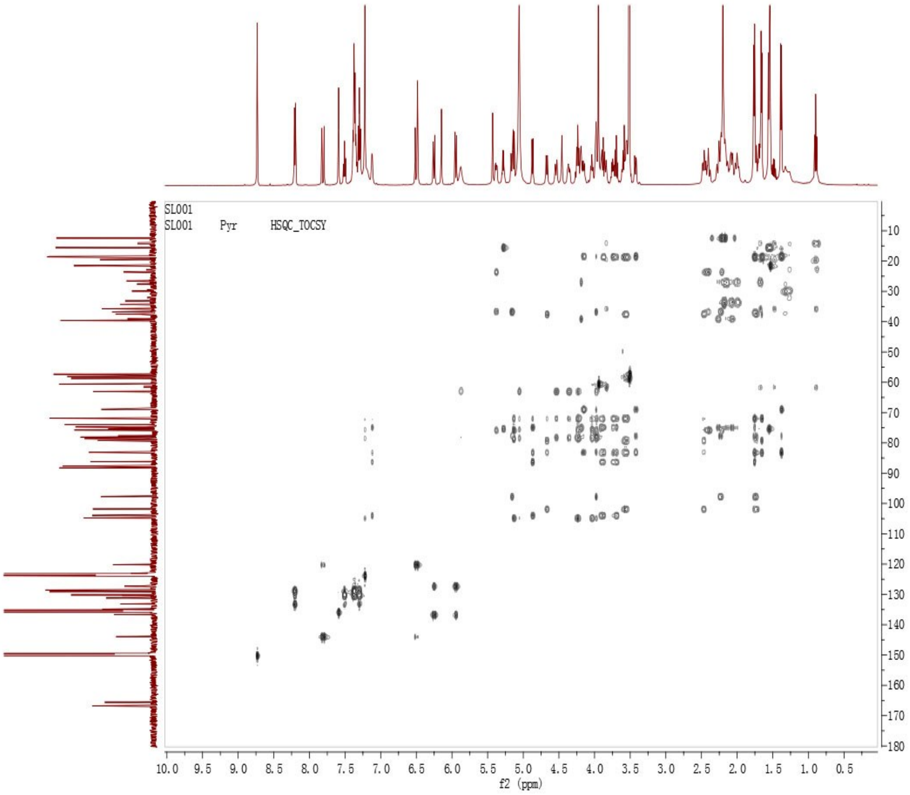


**Figure S41.** HMQC-TOCSY spectrum of **5** in Pyridine-$d_5$.

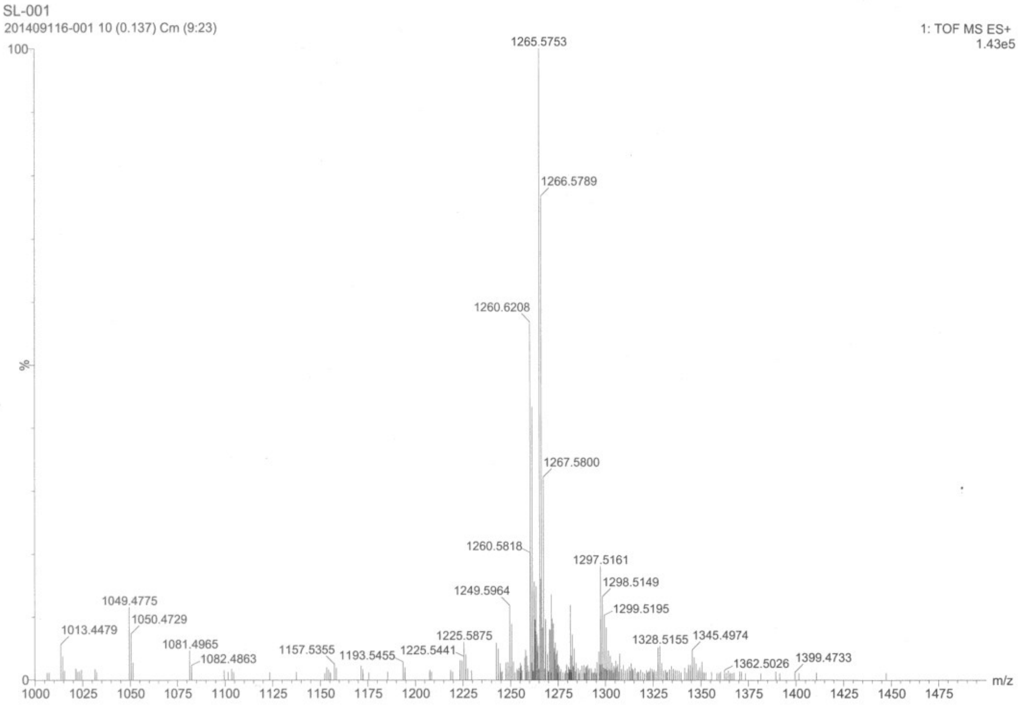


**Figure S42.** HRESIMS spectrum of **5**.

**Table S1.** $^{13}$C-NMR (100 MHz) of the aglycones of compounds **1**–**3** and gymnepregosides E, A, C (in Pyridine-$d_5$).

| NO. | δC | | | | | |
|---|---|---|---|---|---|---|
| | **1** [(a)] | **gymnepregosides E** | **2** | **gymnepregosides A** | **3** | **gymnepregosides C** |
| **1** | 27.6 (t) | 27.6 | 27.5 (t) | 27.7 | 27.6 (t) | 27.9 |
| **2** | 26.5 (t) | 26.6 | 26.5 (t) | 26.5 | 26.5 (t) | 26.9 |
| **3** | 74.9 (d) | 74.9 | 74.9 (d) | 74.8 | 74.9 (d) | 75.0 |
| **4** | 39.0 (t) | 39.1 | 39.0 (t) | 39.1 | 39.0 (t) | 39.3 |
| **5** | 74.7 (s) | 74.8 | 74.7 (s) | 74.7 | 74.8 (s) | 75.0 |
| **6** | 136.7 (d) | 136.7 | 136.6 (d) | 136.7 | 136.1 (d) | 136.3 |
| **7** | 127.3 (d) | 127.3 | 127.3 (d) | 127.4 | 127.6 (d) | 127.7 |
| **8** | 74.0 (s) | 74.0 | 74.0 (s) | 74.0 | 73.8 (s) | 74.1 |
| **9** | 36.6 (d) | 36.9 | 36.5 (d) | 37.0 | 36.6 (d) | 37.1 |
| **10** | 39.6 (s) | 39.6 | 39.6 (s) | 39.6 | 39.6 (s) | 39.9 |
| **11** | 23.6 (t) | 23.7 | 23.7 (t) | 23.6 | 23.6 (t) | 24.1 |
| **12** | 75.8 (d) | 75.8 | 75.6 (d) | 75.9 | 75.9 (d) | 76.1 |
| **13** | 58.1 (s) | 58.1 | 57.9 (s) | 58.0 | 58.1 (s) | 58.3 |
| **14** | 88.2 (s) | 88.3 | 88.1 (s) | 88.1 | 88.9 (s) | 89.1 |
| **15** | 33.1 (t) | 33.2 | 33.1 (t) | 33.1 | 33.3 (t) | 33.6 |
| **16** | 34.3 (t) | 34.3 | 34.2 (t) | 34.2 | 33.5 (t) | 33.8 |
| **17** | 87.7 (s) | 87.7 | 87.7 (s) | 87.8 | 87.9 (s) | 88.1 |
| **18** | 12.4 (q) | 12.5 | 12.3 (q) | 12.1 | 12.7 (q) | 12.9 |
| **19** | 21.5 (q) | 21.5 | 21.5 (q) | 21.4 | 21.6 (q) | 21.9 |
| **20** | 75.3 (d) | 78.4 | 74.4 (d) | 74.4 | 70.4 (d) | 70.7 |
| **21** | 15.6 (q) | 15.6 | 15.5 (q) | 15.5 | 19.6 (q) | 19.9 |
| **Cinnamoyl moiety** | | | | | | |
| **1'** | 166.8 (s) | 166.8 | 166.7 (s) | 166.8 | 167.0 (s) | 167.1 |
| **2'** | 120.1 (d) | 120.2 | 120.3 (d) | 120.4 | 119.6 (d) | 119.8 |
| **3'** | 143.9 (d) | 144.0 | 143.7 (d) | 143.8 | 145.2 (d) | 145.4 |
| **4'** | 134.8 (s) | 134.9 | 134.8 (s) | 134.9 | 134.9 (s) | 135.1 |
| **5',9'** | 128.5 (d) | 128.6 | 128.5 (d) | 128.6 | 128.6 (d) | 128.7 |
| **6',8'** | 129.1 (d) | 129.1 | 129.2 (d) | 129.3 | 129.2 (d) | 129.4 |
| **7'** | 130.4 (d) | 130.4 | 130.5 (d) | 130.5 | 130.5 (d) | 130.6 |
| **(*E*)-2-Methyl-2-butenoyl or benzoyl moiety** | | | | | | |
| **1''** | 165.6 (s) | 165.5 | 166.7 (s) | 166.8 | | |
| **2''** | 131.2 (d) | 131.2 | 129.4 (s) | 129.5 | | |
| **3''** | 130.2 (d) | 130.2 | 137.7 (d) | 137.7 | | |
| **4''** | 128.7 (d) | 128.8 | 14.1 (q) | 14.0 | | |
| **5''** | 133.2 (d) | 133.2 | 12.2 (q) | 12.3 | | |
| **6''** | 128.7 (d) | 128.8 | | | | |
| **7''** | 130.2 (d) | 130.2 | | | | |

[(a)] Measured at 125 MHz.
